# Healthcare organization policy recommendations for the governance of surgical innovation: review of NHS policies

**DOI:** 10.1093/bjs/znac223

**Published:** 2022-07-30

**Authors:** Sian Cousins, Hollie S Richards, Jez Zahra, Harry Robertson, Johnny A Mathews, Kerry N L Avery, Daisy Elliott, Natalie S Blencowe, Barry Main, Robert Hinchliffe, Adrian Clarke, Jane Blazeby

**Affiliations:** National Institute for Health Research Bristol Biomedical Research Centre Surgical Innovation Theme, Bristol Centre for Surgical Research, Bristol Medical School, University of Bristol, Bristol, UK; National Institute for Health Research Bristol Biomedical Research Centre Surgical Innovation Theme, Bristol Centre for Surgical Research, Bristol Medical School, University of Bristol, Bristol, UK; National Institute for Health Research Bristol Biomedical Research Centre Surgical Innovation Theme, Bristol Centre for Surgical Research, Bristol Medical School, University of Bristol, Bristol, UK; National Institute for Health Research Bristol Biomedical Research Centre Surgical Innovation Theme, Bristol Centre for Surgical Research, Bristol Medical School, University of Bristol, Bristol, UK; National Institute for Health Research Bristol Biomedical Research Centre Surgical Innovation Theme, Bristol Centre for Surgical Research, Bristol Medical School, University of Bristol, Bristol, UK; National Institute for Health Research Bristol Biomedical Research Centre Surgical Innovation Theme, Bristol Centre for Surgical Research, Bristol Medical School, University of Bristol, Bristol, UK; National Institute for Health Research Bristol Biomedical Research Centre Surgical Innovation Theme, Bristol Centre for Surgical Research, Bristol Medical School, University of Bristol, Bristol, UK; National Institute for Health Research Bristol Biomedical Research Centre Surgical Innovation Theme, Bristol Centre for Surgical Research, Bristol Medical School, University of Bristol, Bristol, UK; University Hospitals Bristol and Weston NHS Foundation Trust, Bristol, UK; National Institute for Health Research Bristol Biomedical Research Centre Surgical Innovation Theme, Bristol Centre for Surgical Research, Bristol Medical School, University of Bristol, Bristol, UK; University Hospitals Bristol and Weston NHS Foundation Trust, Bristol, UK; National Institute for Health Research Bristol Biomedical Research Centre Surgical Innovation Theme, Bristol Centre for Surgical Research, Bristol Medical School, University of Bristol, Bristol, UK; North Bristol NHS Trust, Bristol, UK; University Hospitals Bristol and Weston NHS Foundation Trust, Bristol, UK; National Institute for Health Research Bristol Biomedical Research Centre Surgical Innovation Theme, Bristol Centre for Surgical Research, Bristol Medical School, University of Bristol, Bristol, UK

## Abstract

**Background:**

The governance for introducing innovative surgical procedures/devices differs from the research requirements needed for new drugs. New invasive procedures/devices may be offered to patients outside of research protocols with local organization oversight alone. Such institutional arrangements exist in many countries and written policies provide guidance for their use, but little is known about their scope or standards.

**Methods:**

One hundred and fifty acute NHS trusts in England and seven health boards in Wales were systematically approached for information about their policies. A modified framework approach was used to analyse when policies considered new procedures/devices to be within local organization remit and/or requiring research ethics committee (REC) approval.

**Results:**

Of 113 policies obtained, 109 and 34 described when local organization and REC approval was required, respectively. Procedures/devices being used for the first time in the organization (*n* = 69) or by a clinician (*n* = 67) were commonly within local remit, and only 36 stated that evidence was required. Others stated limited evidence as a rationale for needing REC approval (*n* = 13). External guidance categorizing procedures as ‘research only’ was the most common reason for gaining REC approval (*n* = 15). Procedures/devices with uncertain outcomes (*n* = 28), requiring additional training (*n* = 26), and not previously used (*n* = 6) were within the remit of policies, while others recommended REC application in these situations (*n* = 5, 2 and 7, respectively).

**Conclusion:**

This study on NHS policies for surgical innovation shows variability in the introduction of procedures/devices in terms of local oversight and/or need for REC approval. Current NHS standards allow untested innovations to occur without the safety of research oversight and thus a standard approach is urgently needed.

## Teaser

Innovative surgery and devices are important to improve patient care. When being offered to patients this requires research or local NHS oversight to ensure transparency and safety monitoring. This study investigated local NHS policies for the oversight of surgical and device innovation in England and Wales. Detailed document analysis revealed a lack of clarity and consistency about what requires ‘research approval’ and what can be delivered with local NHS oversight. A standardized and clear approach is recommended for the governance of surgical innovation.

## Introduction

Innovation in invasive procedures, including surgery, is important. It is often driven by the desire to improve care and is encouraged^1^. Innovation ranges from completely new (first-in-human) procedures to modifications and adoption of existing techniques. Worldwide, the regulation governing innovation in surgery and invasive procedures/devices differs from the rigorous incremental standards required to introduce and modify pharmaceutical products. In the UK, this is via the Medicines and Healthcare products Regulatory Agency (MHRA), in the USA it is the Food and Drug Administration’s (FDA) Centre for Drug Evaluation and Research, and in the European Union it is the European Medicines Agency (EMA). No such dedicated body or regulation for the development and evaluation of surgical innovations exists. Innovative invasive procedures and devices may be delivered to patients with local oversight via policies implemented by clinical effectiveness committees in the organization^2^. They may also be introduced within formal research studies with research ethics committee (REC) approval, although historically this has been uncommon and innovations in surgery have occurred without formal registration anywhere^3–5^. The distinction between local organization oversight and governance via REC is important as it has implications for patient information provision, and reporting outcomes, harms, and adverse events. Research structures provide a systematic framework for governing the evaluation and introduction of innovative procedures/devices. Under research settings it is required that patients are given written information and provided with a choice about receiving the intervention, adverse events are reported, and an appropriate sponsor who is responsible for the study is identified. Under local hospital governance requirements, it is less clear what standards are necessary and there is little known about existing policies. Limited publications examining these issues are available^2,6^. There are several examples of patient harm caused by procedures and devices being used in clinical practice before evaluation of safety and effectiveness are well known^7–9^. Understanding the remit of local organization policies for the introduction of innovation invasive procedures and devices is therefore important.

Guidance from national and international professional bodies^10–13^ suggests that surgeons refer to local organization policies when introducing new procedures/devices. Although these exist across the NHS and worldwide^2,14^, there has been no previous research found that has systematically or widely examined them. Previous work is limited to case studies of individual hospitals and qualitative investigations of surgeon decision-making in this area^2,6^. It is not known when new invasive surgical procedures/devices are appropriate for introduction via local governance policies, and thus can be delivered to patients with local oversight, or when new procedures require evaluation within formal research studies with REC approval.

The INTRODUCE study^15^ examined NHS organization governance policies for the introduction of new invasive procedures and devices. The aim of this paper is to describe when policies consider the introduction of new procedures/devices to be under local NHS organization clinical effectiveness committee governance remit and when they recommend that REC approval is sought.

## Methods

A content analysis of policy documents using a modified framework approach was undertaken^16,17^. This approach provides a systematic and rigorous approach involving coding of text into descriptive themes to form a framework in an iterative process of constant comparison.

### Sampling and data collection

All acute trusts in England (*n* = 150) and seven health boards in Wales were approached and asked for governance policies for introducing new invasive and surgical procedures between November 2017 and November 2018. Document inclusion criteria are described in the protocol^15^.

### Data extraction and analysis

The content analysis started with careful reading and re-reading of all policies by one reviewer (H.S.R.) to ensure familiarization and an in-depth understanding of the data. Verbatim text describing when ‘new’ invasive procedures/devices could be delivered with local NHS organization governance and when instead REC application was recommended was extracted and imported into a data-management program (Microsoft^®^ Excel, version 2015). This formed two data sets. The number of policies contributing to each were counted. A second reviewer independently extracted data from 20 per cent of policies (S.C., J.B., J.A.M., H.R., and J.Z.), rather than the 10 per cent specified in the protocol^15^ because initial reading revealed very heterogeneous text that required careful categorization. The use of multiple reviewers to extract data ensured that mistakes (e.g. missed data) were minimized and reduced the risk that data selection was influenced by a single-person biases^18^. Disagreements were discussed until consensus was reached.

For each data set, individual themes and subthemes were developed from iterative inductive ‘open’ coding process performed iteratively by three reviewers (H.S.R., S.C., and J.B.). Where coded text could not be allocated to an existing theme, a new theme was created. Overarching themes were created by grouping together similar individual themes. ‘Combined overarching’ themes, linking two overarching themes, were created if descriptions of procedures and devices within local organization remit or requiring REC application contained the conjunction ‘and’, linking two thematically discrete elements. The approach was ‘modified’ in that the content of the policies was examined only with reference to the specific research question (i.e. how policies described ‘new’ procedures and devices within local NHS organization governance remit and those recommended for REC application).

The final frameworks including all coded text for all overarching and combined overarching themes, individual themes, and subthemes were reviewed for a final time (H.S.R., S.C., and J.B.), and then all policies re-read (H.S.R.) to ensure relevant text had been comprehensively extracted and coded.

Descriptive statistics summarized the characteristics of organizations approached and those with policies, including commissioning region, foundation status, and acute trust type, and information about the policy issue date and planned review date/expiry dates were extracted.

### Patient and public involvement

The concept and design stages of the study were informed by two public engagement events, hosted by the surgical innovation theme and the patient and public involvement group of the Bristol Biomedical Research Centre (BRC) on 20 March and 9 November 2019. The first took place at a shopping centre near Bristol University and the second at an annual supporters’ celebration for university alumni. Both events attracted members of the public and provided an opportunity for people to consider innovation in surgery with interactive life-size ‘operation’ games (‘Operation Game’ (Altitude Events, Felbridge, UK) and ‘Beat the Surgeon’ (University of Bristol, Bristol, UK)) and posters describing the issues, alongside members of the BRC dressed in ‘scrubs’. Opportunities to respond to questions around surgical innovation were given, including preferences for undergoing surgery with robotic technology or standard methods, whether information about the surgeon’s experience of a new procedure was required before receiving a new operation, and what was understood by the term ‘innovation’. People more often voted for new operations in place of standard procedures, but raised issues related to safety, levels of previous testing/evaluation, and surgeon experience. Individual feedback on study findings and recommendations described in this paper was provided by two members of the BRC patient and public involvement group. Both patient representatives were surprised at the variance between policies and highlighted how this contrasted with the introduction of new drugs, questioning why there were such differences. Regarding the recommendations made in the paper, they stated that standardization was important and felt it would be beneficial. Concern was expressed about creating barriers to innovation, especially if different processes required more REC applications, which may discourage surgeons from engaging in the process.

## Results

### Response rates, organization characteristics, and policy review dates

**Fig. 1:**
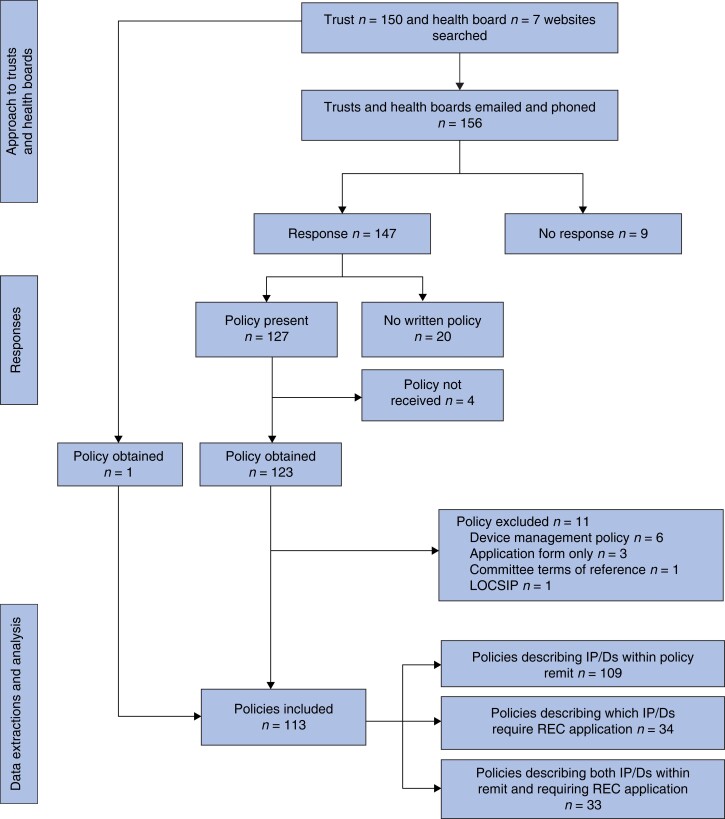
Approach, responses, and policies received from trusts and health boards LOCSIP, local safety standards for invasive procedures; IP, invasive procedures; D, devices; REC, research ethics committee.

Of the 157 organizations approached, 20 said they did not have a policy and nine did not respond (*Fig. 1*). There were 113 policies eligible for inclusion. Policies were received from across all parts of England and three obtained from Wales (*Table 1*). The majority were from foundation status trusts, with policies from all sizes and types (e.g. teaching hospitals) of trust. Fifty policies were out of date (i.e. the planned review date had passed) on receipt and five did not report a planned review date. The average period for planned reviews and updates of policies was 33 months (minimum 12 months, maximum 134 months).

**Table 1:** Characteristics of NHS organizations approached and with included policies for the introduction of invasive procedures and devices

Organization characteristic	Number approached	Number of policies received
**Commissioning region** *		
London	23	15
East of England	18	14
South West	17	14
South East	19	14
Midlands	24	17
North West	26	18
North East & Yorkshire	23	18
Wales	7	3
**Foundation status†**		
Foundation	98	72
Non-foundation	52	38
Acute organization type*,†		
Large	35	26
Medium	32	24
Small	36	22
Multiservice	3	3
Teaching	29	25
Specialist	17	10
**Total**	157	113

* NHS Digital Estates Returns Information Collection, 2018–2019, available from https://digital.nhs.uk/data-and-information/publications/statistical/estates-returns-information-collection/england-2018-19 (accessed 10 November 20).

†England only.

### Overarching and combination overarching themes

Six overarching and seven combined overarching themes were identified (detailed below) from descriptions in policies about when procedures/devices may be introduced via local NHS organization governance (included in 109 policies), and when they instead require REC approval (*n* = 34). All verbatim text coded to all themes is given in the *supplementary material* (*Tables S1 and S2*).

#### Personnel

The expertise and skill of the proceduralist and team was often used by policies to place procedures/devices within the local NHS organization governance remit (*n* = 81; *Table 2*). Most said that procedures being undertaken by a clinician for the first time (*n* = 67) should be brought to the local committee (*Table S3*). Some policies stated that the procedure/device being undertaken by the clinician for the first time would only be within local governance remit if it was also ‘established’ (*n* = 5; i.e. within the *combined overarching theme—Personnel and Evidence*) or being delivered for the first time in the organization (*n* = 3; *combined overarching theme—Personnel and Place* (*Table 2*)). The need for additional training (*n* = 26), delivering the procedure/device in an extended role (*n* = 18), or those personally developed by a clinician for an individual case (*n* = 6) were also specified as being suitable for approval by local NHS committees (*Table S3*). Policies less commonly used the expertise of the proceduralist/team as a rationale for recommending REC application (*Table 3*). Two policies said if the procedure/device required additional training then it should go to a REC (*Table S4*).

**Table 2:** Overarching and combined overarching themes describing new procedures and devices within local NHS policy remit, with number of policies and examples

Overarching and combined overarching themes *Description*	Number of policies (*n* = 109)*	Examples are verbatim policy text
**Personnel** *Limited expertise of procedurists and teams*	81	An interventional procedure should be considered new if […] a doctor no longer in a training post is using it for the first time [policy 037]
**Personnel *and* Evidence**	5	Established procedures which are new to the clinician who is using it for the first time in his or her NHS practice [policy 066]
**Personnel *and* Place**	3	…new to the trust and new to the clinician [policy 136]
**Place** *Delivered for first time in a specific location*	78	Clinicians wishing to introduce into the trust a new clinical technique or procedure not previously undertaken before in the organization must seek approval in accordance with this Policy [policy 012]
**Place *and* Evidence**	31	Techniques and procedures that will form an accepted part of normal clinical practice, whose introduction is supported by a referenced evidence base [policy 032]
**Place *and* Procedure**	3	Any procedure new to the organization (even where it has been practised elsewhere in the NHS), which represents a major change in practice [policy 156]
**Place *and* External guidance**	3	… a ‘New Intervention’ should more usefully be defined as one of the following: One new to the NHS and not already registered with the NICE IPAC group [policy 156]
**Place *and* Economic**	2	It is a new procedure to the organization and has implications relating to cost and/or involvement of other services or professions [policy 154]
**Procedure** *Changes to the procedure itself (including co-interventions) are proposed*	70	A move from open surgery to endoscopic procedure would be new to the trust and would require the completion of a new procedure proposal [policy 016]
**Evidence** *Degree of underpinning evidence*	42	‘First Time’ procedures: they are entirely novel, with an unknown or uncertain efficacy and/or safety profile; or they are a variation of an established procedure which is likely to have a different efficacy and/or safety profile from that of the established procedure [policy 051]
**Economic** *Impact on finance/resource use*	10	Where a change in practice (minor or major in clinical terms) would have financial consequences this must be approved [policy 080]
**External guidance** *Recommendations from NICE or other national/international bodies*	3	A NICE Interventional Procedure that has been approved for use and is no longer within the research specification [policy 098]

NICE, National Institute for Health and Care Excellence; IPAC, Interventional Procedures Advisory Committee.

* Policies may be coded to more than one theme.

**Table 3:** Overarching and combined overarching themes describing new procedures and devices recommended for research ethics committee application, with number of policies and examples

Over-arching and combined over-arching themes *Description*	Number of policies (*n* = 34)*	Examples are verbatim policy text
**External guidance** *Recommendations from NICE or other national/international bodies*	18	When NICE classify the arrangement type of a particular procedures as Research only (use only in the context of a research protocol), the default position of [the committee] will be to refuse the application, and to redirect the applicant to considering the procedure within the context of a research study [policy 001]
**Evidence** *Degree of underpinning evidence*	13	All new procedures and techniques introduced are evidence based or they will form part: of a REC and Trust Research & Development-approved trial [policy 140]
**Evidence *and* External guidance**	2	Those which are not established in clinical practice within the NHS and have not yet been notified to NICE IPAC [policy 092]
**Place** *Delivered for first time in a specific location*	7	The procedure has never before been undertaken anywhere. In this case the proposal is research and should follow standard research governance protocols [policy 025]
**Place *and* Evidence**	1	If it has not previously been used in [the trust/health board] and is not an established technique and/or device: it is the responsibility of applicants to [the committee] to ensure that they have sought appropriate independent review of their proposal and to establish whether an application to a REC is required [policy 049]
**Personnel** *Limited expertise of proceduralists and teams*	2	If the profession considers a procedure or treatment is sufficiently novel as to require special training and assessment before being introduced into clinical practice, then its use should be limited to a number of specified centres for clinical trials [policy 106]
**Procedure** *Changes to the procedure itself (including co-interventions) are proposed*	1	Innovative treatment is only considered research if gene therapy is included [policy 041]

NICE, National Institute for Health and Care Excellence; REC, Research Ethics Committee; IPAC, Interventional Procedures Advisory Committee.

* Policies may be coded to more than one theme.

#### Place

First-time delivery of the procedure/device in a location was used frequently by policies to signal when procedures/devices could be introduced with local governance (*n* = 78; *Table 2*). This was most commonly when it was first-time delivery in the organization (*n* = 69; *Table S3*). Some policies said that procedures/devices being delivered for the first time in the organization were only within the local remit if they: had an evidence base (*n* = 13) or were established in clinical practice (*n* = 10; *combined overarching theme—Place and Evidence*); were significantly different from current practice (*n* = 3; *combined overarching theme—Place and Procedure*); had financial implications (*n* = 2; *combined overarching theme—Place and Economic)* (*Table S3*). Importantly, none of the policies defined these terms. Clinicians wishing to deliver procedures/devices for the first time in the NHS or for the first time anywhere were advised to apply to the local committee for approval, in seven and six policies, respectively. In contrast, seven policies said procedures/devices being used for the first time anywhere should be assessed and approval given by a REC (*Table 3* and *Table S4*). One policy stated if procedures/devices had not been delivered/used in the organization before they should seek REC approval, but only if they were not established techniques/devices (*combined overarching theme—Place and Evidence*). Again, in these policies no definition of what was understood by ‘an established technique’ was provided.

#### Procedure

Proposed changes to components of the procedure/device itself could be introduced via the local approval in 70 policies (*Table 2*). Forty-two said that procedures/devices where major modifications were planned were within their remit, and nine gave examples of what a major modification would constitute (*Table S3*). Procedures/devices being used for a different indication (*n* = 20), in a different part of the body (*n* = 5), or combined with other treatments (*n* = 3) were also described as appropriate for introduction via local governance processes. The need for enhanced or modified consent processes (*n* = 4) or whether different aftercare was required (*n* = 1) was also used as an indication as to whether the procedure or device was within local NHS organization governance remit. Descriptions of how procedures/devices may have changed were not used by any policies to indicate that REC approval should be sought, except in one policy, which only said this was needed if the procedure involves gene therapy (*n* = 1; *Table 3*).

#### Evidence

Just under half of the policies (*n* = 42; *Table 2*) used the degree of underpinning evidence as a reason for the procedure/device to be introduced with local governance. Policies described procedures/devices with ‘uncertain’ or changed outcomes (*n* = 28), those being used in research studies (*n* = 17) or prior to the start of a study (*n* = 1) as being appropriate for introduction with local governance approvals (*Table S3*). No policies expanded on what was meant by uncertain or changed outcomes.

The degree of underpinning evidence was highlighted by 13 of the 34 policies that gave recommendations for when REC approval should be sought (*Table 3*). Policies similarly talked about procedures/devices with ‘insufficient’ evidence (*n* = 6) and ‘uncertain’ or changed outcomes (*n* = 5) but recommended that these required REC approvals, rather than local governance, before delivery in the organization (*Table S4*). Only two policies suggested what level of evidence would be considered ‘insufficient’ (i.e. if the procedure was not in use elsewhere as described in a peer-reviewed publication or if there was no evidence supporting use in nationally or internationally recognized centres; *Table S2*). Four policies used terms like ‘original’, ‘completely new’, and ‘experimental’ to describe procedures/devices requiring REC application, but none defined what was meant by these. Two policies said that if the procedure/device was not established *and* its delivery/use had not been notified to the National Institute for Health and Care Excellence’s (NICE) Interventional Procedures Advisory Committee (IPAC) then REC application was necessary (*combined overarching theme—Evidence and External guidance*).

#### Economic

Ten policies said that where the introduction of procedures/devices had financial implications they should be brought to the committee (*Table 2*). No policies used financial/resource implications as a reason to apply for REC approval.

#### External guidance

Three policies said that procedures/devices recommended for use by NICE were appropriate for introduction by the local policy (*Table 2*). Policies placed more emphasis on external guidance when describing when REC approval should be sought (*Table 3*). Most (*n* = 109) of the 113 policies talked about NICE guidance, including for example, the need to adhere to their recommendations and notify them of procedures, however only 15 policies contained explicit text stating if NICE has classified the procedure/device as to be delivered in ‘research only’ then REC approval should be sought. Similarly, three policies said if there was no national or NICE guidance for the procedure/device it should be evaluated within a research study with REC approval (*Table S4*).

### Inconsistencies across policies

For some procedures/devices conflicting recommendations regarding necessary governance approvals were given across policies (*Table 4*). Twenty-eight stated that procedure or devices with uncertain or changed outcomes were within local NHS policy remit (*Table S3*). However, two of these, along with three different polices, also recommended REC application in these circumstances (*Table S4*). Twenty-six policies said procedures/devices requiring additional training were within local NHS remit, and two others recommended REC application. Finally, six policies stated procedures/devices not previously used anywhere could be introduced via the local NHS organization policy, but seven others recommended REC application.

**Table 4:** Inconsistencies in new invasive procedures/devices within the remit of local NHS policies and those recommended for Research Ethics Committee (REC) application

Overarching theme Individual theme	Number of policies	
	Within local NHS policy remit	REC application recommended
**Evidence** There are uncertain or changed outcomes*	28	5
**Personnel** Delivery/use requires additional training	26	2
**Place** Delivery/use is for the first time anywhere	6	7

* Two policies stated both that new invasive procedures/devices with uncertain or changed outcomes were within local policy remit and that they recommended REC application.

## Discussion

This study has systematically examined policies for the introduction of new invasive procedures and devices into NHS organizations and clinical practice across England and Wales. Specifically, it examined guidance for when local NHS organization oversight and/or REC application is recommended. Policies were obtained from 113 of 157 hospitals approached, with 20 reporting the absence of a written policy. Whereas almost all described when new procedures and devices may be introduced with local organization oversight, only a third gave guidance on when REC application was advised. Detailed analyses identified considerable variation and inconsistencies about what governance (local organization approval *versus* REC) was required, and some common themes were identified. The placement of novel procedures/devices under local governance was largely determined by the experience of the personnel (e.g. if a clinician was delivering it for the first time in the NHS) or whether they were being delivered for the first time in a given location (e.g. first time in the organization). In contrast, recommendations to seek REC approval were mainly based around external guidance (e.g. NICE) and evidence underpinning the procedure. Policies lacked details about required degrees of published evidence to support delivery of a new procedure. Definitions of important terms such as ‘uncertain’, ‘sufficient’, and ‘experimental’ were limited. The ambiguity and inconsistencies observed in the documents highlight an important gap in the governance of innovation in surgery. This has direct implications for patient safety. It means that untested procedures can be introduced and delivered in clinical practice with local NHS oversight alone. Without the framework of research regulation patients may not be informed of the innovative nature of procedures and that may limit their choice. It also means that outcome and safety data may not be collected and reported. Although examples of patient harm caused by innovative procedures and devices are well known and published, the full extent of the problem is unknown^7–9,19^. This analysis of NHS policies identifies the need to improve the governance and regulation of innovation in invasive procedures and surgery.

Worldwide^10,12,13,20^, there are similar approaches to those in the UK for the introduction of innovative surgical procedures. Publications from the USA^21,22^, Australia^23,24^, and Canada^6^ include recommendations for local hospital review if a procedure has significantly changed^6,23^ or if it has not been delivered in the organization^6,24^ or on a large scale^21^ previously. Two of the papers proposed a staged rollout of innovative procedures, overseen by the local committees^21,23^ and one^22^ recommended that interventions that were first in human and/or high-risk procedures, and those with no outcome data to be overseen by research (i.e. an FDA-regulated Institutional Review Board). All the publications and UK guidance lack clarity about when innovations should be delivered within a research study with ethical approval. They are also unclear about what is understood by ‘new’ procedures that fall within local remit. Understanding what surgical innovative interventions require research oversight is therefore important. The Health Research Authority in the UK does define what constitutes research. It says that research involves changing treatment/care services from accepted standards^25^ and stipulates REC approval is required if this involves NHS patients^26^. However, no further detail is given regarding the necessary magnitude or nature of change. It defines research based on its characteristics, such as the use of randomization, and the deliberate use of methods intended to collect quantitative or qualitative data^27^. This is similar to the US Department of Health and Human Services, which defines research as systematic investigations designed to develop or contribute to generalizable knowledge^28^. These definitions of research currently overlap with the recommendations from local hospitals about new procedures making it confusing to surgeon innovators about what oversight is appropriate. The inconsistent approach to governance for the oversight of innovative surgical interventions and devices in surgery is evident in systematic reviews of specific innovations. Reviews of novel techniques in minimally invasive transthoracic liver resection, magnetic augmentation of the lower oesophageal sphincter, and laparoendoscopic colonic polyp resection, for example, show variability in the number of observational and non-comparative studies reporting REC approval (between 25 per cent and 67 per cent)^3–5^. There may be several reasons why this happens. It may reflect historical behaviour and surgeons having different conceptual ideas about what is innovation and research. Although others have highlighted challenges in defining surgical innovation^29^, the conceptual ideas are considered to be similar. Innovations in surgery have uncertain risks. If there are uncertainties about a new procedure this requires transparency with patients and additional monitoring. This is what is provided within a research setting. Although it can be challenging to determine what magnitude of innovation is within some degree of ‘normal variation’, the benefits of designing and delivering early-phase case studies to examine risks and outcomes of innovative procedures are important. They may be used to examine technical modifications, optimal patient groups, and safety outcomes, even in small groups of patients^30^. This approach would facilitate innovation conducted within ethically approved protocols where patients are informed about the innovative nature of the surgery and given the choice to undergo this or standard surgery. Protocols would ensure that safety and technical outcomes are collected systematically and shared (to aid other innovators). In the UK, NICE IPAC provides recommendations for procedures not yet established in clinical practice and established procedures where new evidence has called into question its effectiveness or safety^31^. They often recommend such procedures are undertaken within research settings or otherwise. It is unknown to what extent innovators follow NICE IPAC recommendations or how frequently innovators notify NICE IPAC so that guidance may be developed. Earlier studies show that there are barriers to the implementation of guidance^32,33^, and none of the guidance is mandated.

The strengths of this report include the novelty in examination of these documents and the good response rate. Only nine of 157 organizations did not respond, reflecting the rigorous approach. This study is also detailed and the verbatim text policy content was analysed exhaustively using an in-depth qualitative approach. Text was coded by a second reviewer and there was extensive iteration between throughout analysis. All extracted and coded data are available in *Table S1* and S2 for transparency. The study does have weaknesses: it is possible that organizations may have updated policies since this work was undertaken. Policy update review periods vary; however, over a third of policies received were technically ‘out of date’, and so it is unclear to what extent policies are updated regularly. It is unlikely that organizations updated these during the COVID-19 pandemic. This potential weakness must be balanced with the rigorous analysis conducted. The degree to which policies are practically utilized could not be ascertained by this study. If policies are implemented to the letter, there is huge variability across organizations. If practice differs from that outlined in policies, it would not be inappropriate to assume that the picture may, in fact, be worse. Finally, the current study only examined UK NHS policies. Given the lack of surgery-specific regulatory agencies worldwide, the inconsistencies found are likely to be mirrored in other healthcare systems.

The variation, lack of clarity, and contradictions identified across policies for the introduction of innovative procedures has, for the first time, identified a major risk within NHS governance systems. The development of standardized national guidance for surgeons and clinicians introducing new and modified invasive procedures is essential. Recommendations for improvement are echoed by the 2020 Cumberlege report^34^, which highlighted the disjointed nature of guidance across healthcare bodies. Guidance needs to have clarity about what procedures can be approved by local hospitals and what requires research ethics oversight. It should define clearly what magnitude of ‘novelty’ of a procedure/modification requires research approval, and what constitutes ‘sufficient evidence’ to allow a procedure or device to be used with local oversight, without ethical approval. Additional ways of managing patient information provision and outcome monitoring could be outlined when this is the case. Possible methods for monitoring outcomes could include the mandated use of registries^35^. It is the recommendation of the authors that local organizations only approve procedures with supporting published evidence of safety and efficacy. In these instances, it is possible that clinician and team training are sufficient for delivery. The authors also consider that local processes for registering the procedure and following up on patient outcomes need to be more rigorous. Work to make research processes for overseeing early-phase studies of surgical innovation more efficient is needed and clinicians require support to engage with these processes meaningfully. Informed debate and work with key stakeholders is underway. Ultimately, it is hoped that the provision of clearer guidance in all these matters will support innovation and safeguard patients, surgeons, and the healthcare providers.

## Supplementary Material

znac223_Supplementary_Data

## Data Availability

The study protocol has been published^15^ and all analysed data are provided in the *Supplementary material*.

## Notes

*Disclosure.* The authors declare no conflict of interest.
