# Supplementary material for: Healthcare organization policy recommendations for the governance of surgical innovation: review of NHS policies
Source: Br J Surg. 2022 Jul 30;109(10):1004–12. doi: 10.1093/bjs/znac223 (PMC10364689; doi:10.1093/bjs/znac223)
Supplement: znac223_Supplementary_Data [file znac223_supplementary_data.zip › Supplementary Table 1_Final.docx]

Supplementary Table 1. Verbatim policy text coded to themes describing when new invasive procedures and devices are within local NHS policy remit

| **Over-arching theme**  Individual theme  Sub-theme  *Verbatim policy text* | **Number of policies with text coded to theme, n=109^1^** |
| --- | --- |
| **Personnel** | **81** |
| When the individual clinician will deliver/use the invasive procedure/device for the ‘first time’: | 67 |
| … anywhere, and the policy does not state whether it is already in use elsewhere  ***Policy 010****: The Policy explains what clinicians need to do prior to performing an interventional procedure that they have not used before*  ***Policy 013****: Any doctor / clinical staff considering use in the NHS/[the trust/health board] of a new interventional procedure which he/she has not used before, […] should seek the prior approval of the [the committee].*  ***Policy 014****: a new interventional procedure that they have not used before […] new interventional procedure in [the trust/health board], that they have not used before,*  ***Policy 017****: All medical devices and products, including trials, whether new to […] the individual clinician must be subject to governance approval prior to use. […] Medical device related procedures, products and practice whether new to the […] individual clinician […] or to the individual clinician*  ***Policy 019****: Before introducing a new procedure (whether new to the individual)*  ***Policy 037****: New interventional procedure. An interventional procedure should be considered new if either a doctor no longer in a training post is using it for the first time*  ***Policy 039****: A procedure, intervention or technique (excluding medicines) which is new […] to an individual clinician*  ***Policy 055****: Clinicians wishing to introduce a new interventional procedure to [the trust/health board] that they have not performed before*  ***Policy 056****: New Interventional Procedures and/or Devices may be: (c) New to the particular practitioner*  ***Policy 060****: New interventional procedures are those that are: […] not previously performed by the clinician*  ***Policy 061****: a clinician who is considering performing a procedure new to them*  ***Policy 063****: has not previously been undertaken by an individual member of staff*  ***Policy 068****: New to the person performing [the] procedure*  ***Policy 071****: the use of a new interventional procedure that he/she has not used before […] It also sets out the need for any medical practitioner planning to carry out a procedure new to either the individual*  ***Policy 080****: Introducing new technologies and procedures. Procedures […] new to the individual clinician.*  ***Policy 081****: any [the trust/health board] consultant or senior member of the clinical staff who wishes to adopt, develop or apply to use an Invasive Clinical Procedure new to the consultant,*  ***Policy 082****: the use of a technique for the first time […] by an individual within [the trust/health board] across all professional and non-professional staff groups […] Any doctor considering the use in the NHS of a new interventional procedure which he/she has not used before.*  ***Policy 088****: Clinicians wishing to introduce a new IP that they have not performed before*  ***Policy 095****: The Policy also applies to clinicians new to [the trust/health board] who wish to perform a new Interventional Procedure which he or she has not used before*  ***Policy 096****: The Policy relates to the introduction of procedures, techniques (including laboratory tests), and therapies that are new […] to individuals employed by [the trust/health board].*  ***Policy 098****: An interventional procedure should be considered new if a doctor no longer in a training post is using it for the first time in his/her clinical practice*  ***Policy 106****: Those which are new to a particular operator (performing the procedure for the first time in [the trust/health board] […] Procedures or treatments which are new to a particular operator performing the procedure for the first time [..] Any clinician wishing to introduce a new procedure, treatment or new clinical skill which they have not undertaken before*  ***Policy 107****: A procedure/technique is also considered new if a clinician no longer in a training post is using it for the first time in their clinical practice*  ***Policy 120****: Clinicians wishing to introduce a new interventional procedure which he/she has not used before*  ***Policy 121****: An interventional procedure should be considered new if a clinician no longer in a training post is using it for the first time in his or her clinical practice (HSC 2003/011).*  ***Policy 122****: An interventional procedure should be considered wholly new if either a doctor no longer in a training post is using it for the first time*  ***Policy 124****: Any clinician considering using a new interventional procedure which he/she has not used before,*  ***Policy 133****: An interventional procedure should be considered new if either a doctor or other professional no longer in a training post is using it for the first time […] Is the procedure new to the requesting clinician(s)?*  ***Policy 134****: It has not previously been undertaken by an individual member of staff*  ***Policy 148****: it has not previously been undertaken by an individual member of staff.*  ***Policy 153****: performing a new interventional procedure in the NHS which he/she has not done before* | 31 |
| … anywhere, and the procedure/device may be in use by other clinicians in the NHS or the organisation  ***Policy 033****: if the procedure is new to the practitioner but not [the trust/health board] […] This Policy will apply to any clinician wishing to undertake new clinical interventional techniques or procedures, which may or may not have been performed within [the trust/health board] before*  ***Policy 054****: A procedure new to a doctor no longer in training, even if the procedure has been carried out in [the trust/health board] before.*  ***Policy 065****: if a clinician proposes to start using a procedure for the first time, even if other colleagues in the same department are already doing so.*  ***Policy 092****: This Policy is concerned with four types of procedures: […] if the procedure is new to the practitioner but [the trust/health board].*  ***Policy 113****: This procedure sets out what should be done if a procedure is new to the consultant, but not new to [the trust/health board] (i.e. colleagues are already using the procedure, and the individual consultant wishes to take it up too)*  ***Policy 114****: This Policy covers not only treatments […] or techniques that are new to the practitioner, even if such are already available from other practitioners within [the trust/health board]. […] a technique that is either new to him or herself*  ***Policy 119****: This Policy covers the introduction of techniques new […] to the individuals, regardless of whether or not they may be commonplace elsewhere within the NHS.*  ***Policy 126****: For the purposes of this Policy it is interpreted as: A procedure that a clinician has not used before […] In the case of a doctor newly appointed to [the trust/health board], or a procedure that the doctor has not used before and who wishes to undertake a procedure, even if widely used in the NHS.*  ***Policy 136****: new to the clinician but already established within [the trust/health board].* | 9 |
| … in the organisation, and the ‘competent training clinician’ is not present  ***Policy 052****: clinicians beginning to undertake procedures which are new to them but are currently being undertaken by others within [the trust/health board] (“new operators”) where the competent training clinician is not present to supervise.* | 1 |
| … in their NHS clinical practice, but the clinician may have done one or used one outside the organisation  ***Policy 003****: In this context a practice will be considered new if a doctor, no longer in a training post, is using it for the first time in his or her NHS clinical practice.*  ***Policy 004****: NICE considers an interventional procedure to be "new" if a fully trained clinician is considering the use of the procedure/technique for the first time in the NHS outside of a Research Ethics Committee approved protocol*  ***Policy 010****: An interventional procedure should be considered new if a practitioner no longer in a training post is using it for the first time within NHS clinical practice*  ***Policy 013****: Any doctor/clinical staff considering use in the NHS/[the trust/health board] of a new interventional procedure which he/she has […] only used outside the NHS […] A doctor no longer in a training post is using it for the first time in his or her NHS clinical practice*  ***Policy 014****: a new interventional procedure that they […] have only used outside the […] NHS*  ***Policy 018****: Any doctor/clinician considering use in the NHS of a new interventional procedure which he/she has […] only used outside the NHS*  ***Policy 019****: An interventional procedure must be considered new if a doctor no longer in a training post is using it for the first time in his or her NHS clinical practice (HSC 2003/011)*  ***Policy 021****: the clinicians who will use it are not in training posts and will be performing it for the first time in their NHS clinical practice […] A clinical procedure and interventional procedure should be considered as “new” if a clinician is no longer in a training post and is using it for the first time in his or her NHS clinical practice at [the trust/health board].*  ***Policy 022****: An interventional procedure should be considered new if a health professional no longer in a training post is using it for the first time in his or her NHS clinical practice.*  ***Policy 025****: An interventional procedure should be considered new if a doctor no longer in a training post is using it for the first time at [the trust/health board] in his or her NHS clinical practice. […] Even if they have used it outside of the NHS, they will still have to follow the programme.*  ***Policy 026****: An interventional procedure is considered new if a health care practitioner is no longer in a training post and is using it for the first time in his or her NHS clinical practice*  ***Policy 030****: An interventional procedure should be considered new if a doctor no longer in a training post is using it for the first time in his/her NHS clinical practice.*  ***Policy 036****: The Trust is committed to ensuring that the introduction of new interventional procedures is based on the Interventional Procedures Programme (HSC 2003/011) which outlines the requirements for medical practitioners (post training) planning to undertake a new interventional procedure, which they have not used in the NHS. Even if they have used it outside of the NHS, they will still have to follow the programme. […] An interventional procedure should be considered new if a doctor no longer in a training post is using it for the first time in his or her NHS clinical practice*  ***Policy 038****: An interventional procedure should be considered “new” if a doctor no longer in a training post is using it for the first time in his or her NHS clinical practice.*  ***Policy 039****: ‘New’ Interventional Procedure: An interventional procedure should be considered new if a doctor no longer in a training post is using it for the first time in his or her NHS clinical practice*  ***Policy 043****: An interventional procedure should be considered new if a clinician, no longer in a training post, is using it for the first time in their NHS clinical practice*  ***Policy 048****: A clinician no longer in a training post is using it for the first time in his or her NHS clinical practice. (DoH 2003)*  ***Policy 050****: any clinical practitioner, no longer in a training post, is using a new procedure for the first time in his or her NHS clinical practice*  ***Policy 052****: An interventional procedure should be considered new if a health professional is using it in the Trust for the first time in his or her NHS clinical practice.*  ***Policy 054****: An interventional procedure should be considered new if a clinician no longer in a training post is using it for the first time in his or her NHS clinical practice*  ***Policy 055****: An interventional procedure new to the clinician in his or her NHS clinical practice […] Clinicians wishing to introduce a new interventional procedure to [the trust/health board] that they have not performed before or have only performed outside […] the NHS must comply with the procedure described below.*  ***Policy 056****: It is considered new, if a doctor, no longer in a training post, is using the procedure for the first time in his/ her NHS clinical practice.*  ***Policy 060****: New interventional procedures are those that are: only performed by the clinician outside the NHS*  ***Policy 061****: NICE considers an interventional procedure to be “new” if a fully trained clinician is considering the use of a procedure/technique for the first time in the NHS outside Research Ethics Committee approved protocol.*  ***Policy 064****: An interventional procedure should be considered new is a clinician no longer in a training post is using it for the first time in his or her NHS clinical practice.*  ***Policy 065****: If a Clinician no longer in a training post is using it for the first time in their NHS practice.*  ***Policy 071****: It is considered new if: a clinical practitioner no longer in training is using it for the first time in his or her NHS clinical practice […] the use of a new interventional procedure that he/she […] has only used outside the NHS,*  ***Policy 082****: Any doctor considering the use in the NHS of a new interventional procedure which he/she has not used before, or only used outside the NHS, should seek prior approval. […] the use of a technique for the first time […] by an individual within [the trust/health board]*  ***Policy 088****: New Interventional Procedure: An interventional procedure new to the clinician in his or her NHS clinical practice […] Clinicians wishing to introduce a new IP that they […] have only performed outside the NHS must comply with the procedure described below*  ***Policy 096****: This includes (but is not restricted to) interventional procedures covered by HSC 2003/011 […] For intervention procedures it also applies to clinicians no longer in a training post using a procedure for the first time in their NHS clinical practice.*  ***Policy 100****: Any interventional procedure should be considered new if a doctor no longer in a training post is using it for the first time in his or her NHS clinical practice.*  ***Policy 104****: An interventional procedure should be considered new if: a doctor no longer in a training post is using it for the first time in his or her NHS clinical practice*  ***Policy 105****: An interventional procedure should be considered new if: A doctor not in a training post is using it for the first time in his or her NHS clinical practice (HSC 2003/011)*  ***Policy 113****: This procedure should be considered by a doctor no longer in a training post, who is using a procedure for the first time in his or her NHS clinical practice. (For doctors still in training, the usual procedures for training in new techniques apply.)*  ***Policy 118****: An interventional procedure should be considered new if a doctor, no longer in a training post, or any other staff member in an extended role, is using it for the first time in his or her NHS clinical practice outside formal research. (HSC 2003/011)*  ***Policy 120****: Clinicians wishing to introduce a new interventional procedure […] that he/she has only used outside the […] the NHS*  ***Policy 124****: Any clinician considering using a new interventional procedure which he/she […] has only used outside the NHS*  ***Policy 125****: the healthcare professional, no longer in a training post, is using it for the first time in their NHS clinical practice within this Trust*  ***Policy 126****: For the purposes of this Policy it is interpreted as: A procedure that a clinician […] has only used outside the NHS/Trust. […] new IPs that they have not done before within the NHS*  ***Policy 130****: An interventional procedure should be considered new if a doctor no longer in a training post is using it for the first time in his or her NHS clinical practice*  ***Policy 137****: A ‘new operator’ is a clinician, no longer in a training post, who is using a procedure for the first time in their NHS clinical practice*  ***Policy 138****: An interventional procedure should be considered new if a doctor no longer in a training post is using is for the first time in his or her NHS clinical practice.*  ***Policy 150****: A doctor or independent practicing Healthcare Practitioner (HCP) is no longer in a training post and is using it for the first time in his or her NHS clinical practice.*  ***Policy 153****: NICE considers an interventional procedure to be “new” if a fully trained clinician is considering the use of a procedure/technique for the first time in the NHS outside Research Ethics Committee approved protocol […] performing a new interventional procedure in the NHS which he/she has [performed] […] only outside the NHS,*  ***Policy 156****: An interventional procedure should be considered New if a doctor no longer in a training post is using it for the first time in his or her NHS clinical practice.* | 45 |
| … in the organisation, although the clinician may have done one or used it outside of the organisation  ***Policy 004****: new if any clinician or group is using it for the first time in [the trust/health board].*  ***Policy 013****: Any doctor/clinical staff considering use in the NHS/[the trust/health board] of a new interventional procedure which he/she has not used before […] or only used outside [the trust/health board]*  ***Policy 014****: a new interventional procedure that they […] only used outside [the trust/health board] […] This Policy applies to any clinical staff planning to undertake a new interventional procedure in [the trust/health board] that they have not used before,*  ***Policy 042****: New procedures include […] those which the proposing surgeon may have used during their career at other hospitals*  ***Policy 048****: A clinician no longer in a training post is using it for the first time within [the trust/health board], even if the doctor has performed it in his or her employment in another organisation*  ***Policy 055****: Clinicians wishing to introduce a new interventional procedure to [the trust/health board] that they […] have only performed outside of [the trust/health board]*  ***Policy 095****: The Policy also applies to clinicians new to [the trust/health board] who wish to perform a new Interventional Procedure which he or she has […] only used outside [the trust/health board].*  ***Policy 102****: An interventional procedure should be considered new if a health professional, no longer in a training post, is using it in [the trust/health board] for the first time.*  ***Policy 120****: Clinicians wishing to introduce a new interventional procedure […] that he/she has only used outside [the trust/health board] or the NHS*  ***Policy 126****: For the purposes of this Policy it is interpreted as: A procedure that a clinician […] has only used outside the NHS/[the trust/health board].* | 10 |
| Delivery/use requires additional training | 26 |
| … Additional training is required  ***Policy 010****: A Modification to an interventional procedure is defined as one that could have a staff or clinical team training requirement*  ***Policy 019****: Further, a ‘new’ procedure represents a substantial change in practice in terms of: expertise required to successfully perform the procedure*  ***Policy 021****: The definition of a New Clinical Procedure does not cover […] Minor modifications to existing techniques, not requiring additional training*  ***Policy 023****: It should be noted specifically that [the committee] should review all proposals from clinicians who wish to extend their clinical activity to include techniques for which they had not been trained during their period of formal specialist training.*  ***Policy 025****: “New” means completely novel or a sufficient adaptation of a technique that makes it different in such a way that [..] new training (not re-training) has been required.*  ***Policy 027****: Will the clinical sponsor and other new users require significant training? ‘Significant’ training might involve the potential user attending at a course or visiting an established user at another site (NHS or otherwise)*  ***Policy 041****: For the purpose of this Policy significant change or new procedure which is deemed to be “any clinical change that has an impact on either […] an individual’s training needs, others’ training needs*  ***Policy 043****: A modification to an interventional procedure is defined as one that could have a staff or clinical team training requirement […] planning a major transformation of existing, accepted procedures and practice*  ***Policy 051****: being of significant clinical risk and requiring formal training and assessment of competence in addition to the healthcare professional’s existing practice qualifications. […] Excludes: Minor alterations of existing techniques, procedures or equipment unless they […] require re-training and re-assessment of competence and effectiveness*  ***Policy 064****: Would the introduction of this new technology require significant additional training for those using it and/or for subsequent patient care? This includes, but it not limited to, technologies where arrangements are made for mentoring/proctoring) […] Does the introduction of this new technology represent a significant deviation from what is routine practice or is additional to what is routine? (as opposed to introducing an alternative replacement product that requires no training and carries no additional risk) […] Technology which represents a significant development of existing technology*  ***Policy 065****: However if the new procedure or equipment could reasonably be regarded as no more than a minor variation of a current procedure or instrument already in use in the trust, and in particular carries […] no additional training requirements, then consideration by [the committee] may not be needed;*  ***Policy 071****: it is a modification of a procedure significant enough to require special training*  ***Policy 082****: Minor changes to techniques that […] do not represent a significant departure from local practice in terms of the skills […] required for their local implementation do not need to be evaluated fully as above.*  ***Policy 095****: Anything which is significant in the way of a change (expansion or extension) of practice or where new skills are required for established operations or new procedure is included.*  ***Policy 101****: one for which a member of the MDT will need additional training*  ***Policy 110****: A new technique or procedure is one that […] requires mastery of new or changed technology.*  ***Policy 115****: The Policy does not apply to minor incremental changes to existing procedures unless there is a significant […] competency, […] or training issues requiring Quality & Risk Committee and Care Group approval.*  ***Policy 116****: Will the variation require any additional training?*  ***Policy 128****: A new technique is defined as […] one where there is a need to acquire new skills or knowledge for it to be successfully implemented. […] Established procedures using new equipment or consumables are exempt from this Policy unless there are training needs identified that require acquisition of new knowledge or skills.*  ***Policy 131****: Consideration needs to be given to whether […] training is needed;*  ***Policy 136****: The Policy applies to significant change in clinical practice and to all clinical staff. For the purposes of this Policy. 'significant change' is deemed to be 'any change that has an impact on an individual's training needs, other training needs*  ***Policy 150****: Introduction of NIP through research: [the committee] will also consider NIPs that are introduced to [the trust/health board] within a research protocol where there are training implications or those that will subsequently be included on case registers* | 22 |
| … A proctor is required  ***Policy 013****: This includes; modifications of existing techniques […] where an external expert or proctor is required.*  ***Policy 027****: Will company technical representatives be present for initial use? Will an expert preceptor/proctor participate in initial use? […] Will company technical representatives be present for initial use?*  ***Policy 055****: This may be extended to include any new interventional procedure which requires the attendance of an external person at the first use(s) of the intervention. Examples might include a company representative, application specialist, a visiting surgeon or a proctor.*  ***Policy 088****: This may be extended to include any new procedure which requires the attendance of an external person at the first use(s) of the intervention. Examples might include a company representative, application specialist, a visiting surgeon or a proctor*  ***Policy 116****: Do you need a proctor to introduce the variation into clinical practice?*  ***Policy 126****: Visiting experts demonstrating a new method with the intention of teaching Trust staff.* | 6 |
| Being delivered/used by a different or extended clinical role  ***Policy 012****: The application of new or existing proven techniques to be carried out by a specific clinical/professional staff member extending their role.*  ***Policy 016****: New Expansion of Current Scope of Clinical Practice – e.g. an Allied Health Professional (AHP) or nurse wishing to undertake a clinical procedure, intervention or technique usually performed by a doctor and for which the training standards and established competency packages do not exist locally. [..] or one for which established competencies do not exist for non-medical staff.*  ***Policy 018****: The procedure applies to any changes in current procedures where there is a proposal to provide the procedure for the first time under an extended role e.g. by nurse practitioner. A procedure should be considered new if the procedure is to be undertaken for the first time by a non-medical clinician e.g. as part of the development of a Nurse Practitioner role.*  ***Policy 021****: it is an established procedure but will be used in a new way which will involve significant changes to the roles of clinical staff […] Major changes in clinical roles of staff […] Excludes: Minor modifications to existing techniques, not requiring […] significant changes to roles of clinical staff*  ***Policy 026****: Where the new procedure application relates to nursing, midwifery, allied healthcare professionals or healthcare scientist staff undertaking a new procedure that has not previously been undertaken by the profession within [the trust/health board]*  ***Policy 030****: Where the new procedure application relates to nursing or midwifery staff undertaking a new procedure that has not previously been undertaken by the nursing or midwifery profession within [the trust/health board]*  ***Policy 039****: Expanded Practice: An interventional procedure should be considered new if it is to be performed by a new staff group and therefore expands their practice, for instance a nurse performing an interventional procedure which has previously been performed by a doctor, even though the procedure is not new to the hospital. […] This Policy has now been extended to encompass the process to be followed where non-medical staff (no longer in training) wish to extend their practice and undertake a procedure, intervention or technique which is new to that member of staff e.g. a nurse undertaking a procedure, intervention or technique which is usually performed by a doctor.*  ***Policy 041****: Change or a new procedure in clinical practice may be required due to […] or new ways of working (e.g. new role). […] If the change results in the expansion of the role*  ***Policy 048****: the introduction of any new job role (i.e. a role that did not exist in [the trust/health board] previously, or a new role to your department); the introduction of a new clinical procedure/technique into an existing role; the transfer of an existing technique from one staff group to another*  ***Policy 053****: alternatively a new procedure, intervention or technique which is new to the clinician e.g. an existing employee who wishes to develop a new skill and enhance their clinical practice such as: A nurse wishing to undertake a clinical procedure, intervention or technique usually performed by a doctor […] This expansion in practice includes the introduction of new clinical procedures, interventions and or techniques.*  ***Policy 061****: Where nurses midwives and allied health professionals wish to expand the scope of their practice previously performed by other healthcare professionals, this must clearly benefit patient care and improve service delivery.*  ***Policy 065****: The introduction of new roles and techniques or the transfer of existing techniques between different staff groups […] all techniques or procedures whose use is to be extended to a new group of staff (as an extended role, for example). […] A New Practitioner to undertake an established procedure: A New Role*  ***Policy 080****: Where it is proposed that a procedure previously performed by one staff group or individual is to be transferred to become the responsibility of a different staff group or individual the steps in this document should be followed.*  ***Policy 102****: A new way of working is when the skills and practice normally undertaken by one group of health professionals is transferred, after appropriate training, to another group of health professionals.*  ***Policy 106****: These include […] and new expanded roles in Nursing, Midwifery and Professions Allied to Medicine*  ***Policy 115****: The Policy applies to any changes in current procedures where there is a proposal to provide the procedure for the first time under an extended role e.g. by nurse practitioner. A procedure should be considered new if the procedure is to be undertaken for the first time by a non-medical clinician e.g. as part of the development of a Nurse Practitioner role. […] The purpose of this Policy is to outline the process for validating any new interventional procedure undertaken or extension to clinical practice within [the trust/health board].*  ***Policy 118****: An interventional procedure should be considered new if a doctor, no longer in a training post, or any other staff member in an extended role, is using it for the first time in his or her NHS clinical practice outside formal research.*  ***Policy 147****: This may also apply to procedures already done in [the trust/health board] but where a different group of clinicians is going to undertake them e.g. nurses doing endoscopy, radiographers doing x-ray interpretation* | 18 |
| Developed personally by a clinician or for an individual patient/case  ***Policy 041****: Based on existing guidance, procedure carried out on request and specifically tailored for the patient, which does not involve xenotransplantation, and is not carried out in the context of research, but as part of clinical treatment can be considered as innovative therapy*  ***Policy 055****: This Policy sets out the process to be followed by all clinicians at [the trust/health board] wishing to introduce an interventional procedure that is: […] for emergency or compassionate use for named patients*  ***Policy 061****: All new or personally developed clinical procedures,*  ***Policy 088****: Novel Procedures: Divisional Quality Board will consider applications from clinicians who wish to undertake a novel procedure on a named patient. These are considered to be neither research nor routine, but rather, an exceptional, innovative treatment never previously undertaken. // Compassionate use for a named patient: Occasionally, a clinician may propose a new IP for which there is no NICE guidance and limited or no safety and efficacy data. In situations where this is for compassionate use for a named patient*  ***Policy 107****: What is meant by a new procedure/technique? A new or personally developed operation*  ***Policy 153****: All new or personally developed clinical procedures, […] must be agreed before introduction.* | 6 |
| When any senior clinician involved in the procedure will deliver it for the first time  ***Policy 026****: the procedure would also be considered new if it were new to any one of the senior clinicians involved* | 1 |
| **Personnel *and* Evidence** | **5** |
| When the individual clinician will deliver/use the invasive procedure/device for the ‘first time’ anywhere, and the procedure/device is established  ***Policy 016****: An Existing Recognised Procedure: any intervention or technique which is new to the clinician in which the procedure is nationally recognized by NICE.*  ***Policy 043****: undertaking procedures that are established within [the trust/health board] but are new to the individual.*  ***Policy 066****: Established procedures which are new to the clinician who is using it for the first time in his or her NHS practice*  ***Policy 067****: Established procedures which are new to the consultant who is using it for the first time in his or her NHS practice*  ***Policy 108****: This Policy is concerned with three types of procedures: Those which are established in clinical practice within the NHS but are new to […] this practitioner.* | 5 |
| **Personnel *and* Place** | **3** |
| Delivery/use is for the first time by a clinician and for the first time in the organisation  ***Policy 030****: a new clinical procedure to [the trust/health board] that he/she has not used before,*  ***Policy 131****: established interventions or devices currently undertaken or used in other NHS settings but being used for the first time at [the trust/health board] and for the first time by the practitioner. […] by a practitioner to whom the procedure is also new*  ***Policy 136****: new to [the trust/health board] and new to the clinician* | 3 |
| **Place** | **78** |
| Delivery/use is for the first time in the organisation | 69 |
| … but the policy does not state whether it has been done/used or evaluated elsewhere  ***Policy*** ***003****: Any clinician considering using clinical practice that has not previously been used in [the trust/health board]*  ***Policy*** ***004****: A new clinical procedure is any clinical intervention which involves new techniques which have not previously been undertaken by [the trust/health board]*  ***Policy*** ***007****: It has not previously been undertaken at [the trust/health board]*  ***Policy*** ***010****: are not normally performed by the clinical staff of [the trust/health board]*  ***Policy*** ***014****: This Policy applies to any clinical staff planning to undertake new interventional procedure […] that is not currently used in [the trust/health board]*  ***Policy*** ***021****: or will be introducing it to [the trust/health board]*  ***Policy*** ***026****: any clinical intervention which involves new techniques which have not previously been undertaken by [the trust/health board]*  ***Policy*** ***027****: Is the existing technology routinely used at [the trust/health board]?*  ***Policy*** ***037****: or a new procedure is being introduced within [the trust/health board]*  ***Policy*** ***039****: which is new to [the trust/health board]*  ***Policy*** ***043****: introducing clinical procedures, surgical/medical techniques or equipment new to [the trust/health board]*  ***Policy*** ***051****: Techniques, procedures and equipment that are to be used for the first time [the trust/health board], and if not performed in the correct manner, pose a significant risk to the patient or future patients either in terms of injury or other dimensions*  ***Policy*** ***052****: new interventional procedures to [the trust/health board]. It applies to either: a) totally new procedures to [the trust/health board]*  ***Policy*** ***053****: any clinical intervention which involves new techniques which have not previously been undertaken by [the trust/health board]; // which has not previously been undertaken within the organisation.*  ***Policy*** ***055****: This Policy sets out the process to be followed by all clinicians at[the trust/health board] wishing to introduce an interventional procedure that is: new to [the trust/health board]*  ***Policy*** ***060****: new to [the trust/health board]*  ***Policy*** ***061****: All [..] procedures new to [the trust/health board] // a procedure new […] to [the trust/health board]*  ***Policy*** ***063****: has not previously been undertaken at [the trust/health board]*  ***Policy*** ***064****: or technology that is new to [the trust/health board]*  ***Policy*** ***069****: This Policy will ensure there is a systematic approach for evaluating any application to undertake new interventional procedures at [the trust/health board]. It applies to both NICE interventional and Non-NICE interventional procedures that are new to [the trust/health board] […] All clinicians that wish to introduce a surgical or interventional procedure that is not currently in use in [the trust/health board]*  ***Policy*** ***072****: To be introduced or implemented within [the trust/health board]*  ***Policy*** ***082****: to the use of a technique for the first time in an individual in [the trust/health board]*  ***Policy*** ***083****: not previously been undertaken within the organisation. […] which have not previously been undertaken by the [the trust/health board]*  ***Policy*** ***084****: which have not previously been undertaken by the [the trust/health board]. […] The proposed introduction of any new clinical technique or procedure which has not previously been undertaken within the organisation.*  ***Policy*** ***088****: new to [the trust/health board]*  ***Policy*** ***095****: one which has not previously been used in [the trust/health board]*  ***Policy*** ***104****: An interventional procedure should be considered new if: the procedure is new to [the trust/health board]*  ***Policy*** ***105****: The procedure is new to [the trust/health board] // For new interventional procedures not previously undertaken in [the trust/health board]*  ***Policy*** ***107****: One which has not previously been used in [the trust/health board]*  ***Policy*** ***110****: A new technique or procedure is one that is not in practice at [the trust/health board]*  ***Policy*** ***113****: This procedure is of relevance to clinicians proposing to undertake a new interventional procedure, which is new to [the trust/health board]*  ***Policy*** ***114****: This Policy covers not only treatments that are new to the [the trust/health board] […] or to [the trust/health board]*  ***Policy*** ***116****: any new device, instrument or intervention for diagnostic/therapeutic purposes that has not previously been used in [the trust/health board] […] Is the existing technology/procedure being routinely used in [the trust/health board]?*  ***Policy*** ***121****: If the interventional procedure has not previously been used within [the trust/health board]*  ***Policy*** ***122****: or a new procedure is being introduced within [the trust/health board]*  ***Policy*** ***123****: which has not previously been undertaken within the organisation. […] any clinical intervention which involves new techniques which have not previously been undertaken by [the trust/health board]*  ***Policy*** ***125****: the interventional procedure is new to [the trust/health board]*  ***Policy 133****: or a new procedure is being introduced within [the trust/health board]*  ***Policy 134****: It has not previously been undertaken at [the trust/health board]*  ***Policy 139****: techniques, interventions and medications that are new to [the trust/health board]*  ***Policy*** ***144****: introduce to [the trust/health board] a new clinic, clinical technique or clinical procedure*  ***Policy*** ***146****: if it has not been carried out before in [the trust/health board]. […] An interventional procedure should be considered new if it has not been carried out before in [the trust/health board].*  ***Policy*** ***148****: it has not previously been undertaken at [the trust/health board]*  ***Policy*** ***150****: The standards in this document are relevant to: The proposed introduction of any new clinical technique or procedure which has not previously been undertaken within the organisation.* | 44 |
| … and it may have been delivered/used elsewhere  ***Policy 001:*** *the introduction of new interventional procedures within [the trust/health board] […] The Policy applies to interventional procedure offered by [the trust/health board] to NHS patients, irrespective of the location or staff involved, or if the procedure has been reviewed by NICE.*  ***Policy 002:*** *A procedure should be considered new if any clinician or group is using it for the first time in [the trust/health board] […] This Policy sets out the process for the introduction of all interventional procedures that are new to [the trust/health board] […] Note on clinicians new to [the trust/health board]: If a clinician newly employed in [the trust/health board] brings with them a procedure as part of their existing repertoire but which is not previously undertaken here, implications both under this Policy and for the contract must be considered on recruitment.*  ***Policy 012:*** *Clinicians wishing to introduce into [the trust/health board] a new clinical technique or procedure not previously undertaken before in the organisation must seek approval in accordance with this Policy. […] Clinicians wishing to introduce into [the trust/health board] a new clinical technique or procedure not previously undertaken before on the basis that NICE has issued guidance (Interventional Procedure Guidance IPG, or Medical Technology Guidance MTG), must seek prior approval in accordance with this Policy*  ***Policy 015:*** *Through “The Safety and efficacy register of New Interventional Procedures” (SERNIP) NICE effectively decomposes ‘new’ into range of levels by reference to safe implementation. [If] SERNIP definition is: Safety and efficacy established; procedure may be used [and IP/D is] new to [the trust/health board] but has been used & described elsewhere: [the IP/D] requires scrutiny/assessment by [the committee]*  ***Policy*** ***022****: Health professionals wishing to introduce into [the trust/health board], a new procedure/technique not undertaken in [the trust/health board] previously, for which NICE has previously issued guidance should seek approval from [the trust/health board] in accordance with this Policy […] An interventional procedure should also be considered new if it is new to the organisation, even if the clinician has performed it previously elsewhere.*  ***Policy*** ***025****: is using it for the first time at [the trust/health board] […] In use in health care elsewhere but not in [the trust/health board]*  ***Policy*** ***030****: This* ***Policy*** *applies to any clinician planning to undertake a new clinical procedure to [the trust/health board] […] or has only used outside [the trust/health board].*  ***Policy*** ***036****: In use elsewhere but not in [the trust/health board].*  ***Policy*** ***042****: those which have never previously been performed at [the trust/health board] […] May be performed at other hospitals but not currently used at [the trust/health board]*  ***Policy*** ***050****: The procedure is already the subject of NICE guidance but is new to [the trust/health board] […] Clinicians who wish to undertake a procedure [the trust/health board]*  ***Policy*** ***054****: A procedure that has never been performed in [the trust/health board] before, including those that may have been carried out in other [trusts/health boards] or in the private sector. […] Where a Consultant is newly appointed to [the trust/health board] and it is evident that the practitioner is competent to perform a procedure but the procedure is new to [the trust/health board], the same process applies as outlined*  ***Policy*** ***058****: An interventional procedure should be considered New if it is a procedure that has been […] implemented elsewhere but which has never been previously introduced at [the trust/health board]*  ***Policy*** ***065****: If the procedure is performed for the first time in [the trust/health board] even if the Clinician has performed it in their employment in another organisation. […] The committee must approve all techniques or procedures (including the use of novel equipment) that are new to [the trust/health board]*  ***Policy*** ***071****: it has not been carried out in [the trust/health board] before, whether or not it is established elsewhere […] It also sets out the need for any medical practitioner planning to carry out a procedure new to […][the trust/health board] to gain approval*  ***Policy*** ***080****: Procedures either new to [the trust/health board] […] A new procedure in [the trust/health board] irrespective of prior experience elsewhere (including the private sector)*  ***Policy*** ***096****: that are new to [the trust/health board] […] and new procedures and therapies recommended by NICE*  ***Policy*** ***098****: A procedure in use in other Health Institutions, but not in [the trust/health board]*  ***Policy*** ***101****: Techniques new to [the trust/health board] whether or not widely adopted elsewhere,*  ***Policy*** ***115****: The* ***Policy*** *also applies to new Interventional Procedures under NICE Guidelines and new clinical procedures not previously undertaken within [the trust/health board] (i.e. a clinician may have undertaken elsewhere but not at [the trust/health board])*  ***Policy*** ***119****: This* ***Policy*** *covers the introduction of techniques new to [the trust/health board] […] regardless of whether or not they may be commonplace elsewhere within the NHS.*  ***Policy*** ***131****: Procedure new to [the trust/health board] – previously undertaken in another NHS setting but being used in [the trust/health board] for the first time, either by a practitioner used to doing the procedure*  ***Policy*** ***136****: new to [the trust/health board], but not to the clinician*  ***Policy*** ***137****: The procedure is being used for the first time in [the trust/health board] even if the practitioner has been trained in the procedure elsewhere*  ***Policy*** ***138****: Clinicians wising to introduce into [the trust/health board], a new clinical technique or procedure not previously undertaken before on the basis that NICE has issued guidance, or whether it is still in the notification stage […] a new clinical technique or procedure not previously undertaken before in the organisation but used widely throughout the NHS,.*  ***Policy*** ***153****: procedures new to [the trust/health board] and procedures with NICE Guidance must be agreed before introduction.* | 25 |
| Delivery/use is for the first time in the NHS | 7 |
| … but the policy does not state whether it has been delivered/used outside the NHS  ***Policy 056****: New Interventional Procedures and/or Devices may be: Entirely new to the National Health Service*  ***Policy 057****: completely new to the NHS. […] Introducing a new invasive procedure/associated equipment that is not already in use elsewhere within the NHS […] No procedure or equipment that is completely new to the NHS can be introduced at [the trust/health board] without formal approval from [the committee].*  ***Policy 094****: A treatment or technique that has never been used in the NHS before.*  ***Policy 118****: In the case of a new interventional procedure to the NHS: NICE guidance, via the interventional procedures programme, is not available.*  ***Policy 126****: procedures outside of NICE which they have not done within the NHS before […] new IPs that they have not done before within the NHS* | 5 |
| … and it may have been delivered/used previously outside of the NHS  ***Policy 131****: Procedure new to the NHS – procedure only previously […] used in non NHS settings*  ***Policy 154****: It has only previously been used outside the NHS* | 2 |
| Delivery/use is for the first time anywhere  ***Policy 007****: If the procedure has not been performed before, clinicians must have transferable skills that will help to reduce risks and increase the benefit to the patient.*  ***Policy 023****: A novel procedure is one that has not previously been performed anywhere else on humans*  ***Policy 063****: A procedure, method, technique, technology or therapy may be considered new because it: has never been previously applied and/or undertaken*  ***Policy 131****: “New” means any of the following applies […] never previously undertaken or used in any setting*  ***Policy 134****: It has never been previously undertaken;*  ***Policy 148****: A PROCEDURE OR TREATMENT NEVER PREVIOUSLY UNDERTAKEN* | 6 |
| Delivery/use is for the first time in the division, but may be in use elsewhere in the organisation  ***Policy 017****: All medical devices and products, including trials, whether new to the Division or the individual clinician must be subject to governance approval prior to use. This includes introduction or adoption of procedures or products into the Division that may be in use already in other areas of [the trust/health board]. […] Medical device related procedures, products and practice whether new to the division […] The purpose is to ensure that all procedures, products and practice whether new to the division*  ***Policy 036*** *A new procedure that is emerging in [the trust/health board] elsewhere.* | 2 |
| **Delivery/use is for the first time in the United Kingdom**  ***Policy 010****: techniques or procedures which are new to this country*  ***Policy 116****: Is this the first time the variation has been used in the UK?* | 2 |
| **Place *and* Evidence** | **31** |
| Delivery/use is for the first time in the organisation, and it has an evidence base, has been evaluated or undergone clinical trials  ***Policy 013****: An interventional procedure should be considered new if: A procedure that has an evidence base has not been undertaken in [the trust/health board] previously*  ***Policy 018****: All new procedures / techniques introduced into [the trust/health board] will fulfil the following requirements: They will be an accepted part of normal clinical practice, with a referable evidence based*  ***Policy 032****: The Policy covers all new clinical procedures or techniques introduced into [the trust/health board]. These fall into two main categories, namely: Techniques and procedures that will form an accepted part of normal clinical practice, whose introduction is supported by a referenced evidence base;*  ***Policy 044****: A Novel Therapeutic Intervention is defined as any intervention which has previously undergone clinical trial and is considered safe for clinical practice but has not been performed by a clinician (i.e. doctor, nurse, allied healthcare professional, clinical scientist) at [the trust/health board] before.*  ***Policy 056****: New to [the trust/health board] […] These procedures must have been shown to be effective through clinical trials and/or approval by the Royal College or other accredited bodies.*  ***Policy 058****: An interventional procedure should be considered New if it is a procedure that has been evaluated […] elsewhere but which has never been previously introduced at [the trust/health board]*  ***Policy 059****: Novel Therapeutic Interventions […] are those which have previously undergone clinical trials or other forms of systematic evaluation to demonstrate safety and likely or proven efficacy, but which have not yet been used previously at [the trust/health board].*  ***Policy 081****: The procedure has been examined and tested within a recognised and established framework that demonstrates the clinical purpose, clinical efficacy and safety of the procedure […] any [the trust/health board] consultant or senior member of the clinical staff who wishes to adopt, develop or apply to use an Invasive Clinical Procedure new to […][the trust/health board].*  ***Policy 091****: Applications made in relation to this Policy will be based on a body of evidence and so will not be entirely new outside the Trust. […] of any technique or interventional procedure which has not previously been undertaken within [the trust/health board].*  ***Policy 094****: A treatment/technique that is understood to be safe and effective but is new to [the trust/health board]*  ***Policy 106****: Those which are new to [the trust/health board] […] The introduction of new surgical or medical techniques which have not previously been performed in [the trust/health board] […] Procedures or treatments which are new to [the trust/health board] […] When the value of such a procedure or treatment has been shown to be effective through clinical trials and/or approved by the appropriate Royal College or other accrediting body*  ***Policy 119****: All new procedures/techniques introduced into [the trust/health board] will fulfil the following requirements: They will be an accepted part of normal clinical practice, with a referenced evidence base to that effect*  ***Policy 140****: The proposed introduction of any new clinical technique or procedure which has not previously been undertaken within the organisation. […] All new procedures and techniques introduced are evidence based […] New Clinical Procedure: any clinical intervention which involves new techniques which have not previously been undertaken by [the trust/health board]* | 13 |
| Delivery/use is for the first time in the organisation, and it is established in clinical practice elsewhere  ***Policy 016****: involves new techniques which have not previously been undertaken by [the trust/health board]; […] has not previously been undertaken within the organisation […] Any existing recognised intervention or technique which is to be newly performed at [the trust/health board] […] Any existing recognised intervention or technique which is to be newly performed by the clinician at consultant level (although they may performed it previously in a training role under consultant supervision)*  ***Policy 019****: a procedure that has not previously been routinely undertaken at [the trust/health board] […] Clinicians wish to introduce to [the trust/health board], a new procedure which is already established elsewhere.*  ***Policy 033****: Those which are established in clinical practice within the NHS but are new to [the trust/health board] […] This Policy will apply to any clinician wishing to undertake new clinical interventional techniques or procedures, which may or may not have been performed within [the trust/health board] before*  ***Policy 049****: Any technique or medical device that has not been previously used in the [the trust/health board] is classed as new, regardless of whether it has been used elsewhere […] If it has not previously been used in [the trust/health board] but is an established technique and/or device in the UK*  ***Policy 066****: Established procedures which are new to [the trust/health board], even when the clinician is experienced in this procedure*  ***Policy 067****: Established procedures which are new to [the trust/health board], even when the consultant is experienced in this procedure*  ***Policy 092****: This Policy is concerned with four types of procedures: Those which are established in clinical practice within the NHS but are new to [the trust/health board]*  ***Policy 108****: Those which are established in clinical practice within the NHS but are new to [the trust/health board]*  ***Policy 128****: one that has not previously been performed within [the trust/health board] […] new (but established techniques) into this organisation.*  ***Policy 131****: Procedure new to [the trust/health board] – previously undertaken in another NHS setting but being used in [the trust/health board] for the first time, either by a practitioner used to doing the procedure or by a practitioner to whom the procedure is also new […] established interventions or devices currently undertaken or used in other NHS settings but being used for the first time at [the trust/health board] but where the practitioner has undertaken or used the intervention/device elsewhere.* | 10 |
| Delivery/use is for the first time in clinical practice, and it has been delivered/used in a research setting  ***Policy 004****: For the purposes of the programme, NICE considers an interventional procedure to be "new" if a fully trained clinician is considering the use of the procedure/technique for the first time in the NHS outside of a Research Ethics Committee approved protocol.*  ***Policy 061****: NICE considers an interventional procedure to be “new” if a fully trained clinician is considering the use of a procedure/technique for the first time in the NHS outside Research Ethics Committee approved protocol*.  ***Policy 070****: For the purposes of the programme NICE considers a procedure to be ‘new’ if a fully trained clinician is considering the use of the procedure/technique for the first time in the NHS outside a Research Ethics Committee approval.*  ***Policy 076****: An interventional procedure will be considered new if a fully trained clinician is considering the use of the procedure/techniques for the first time in the NHS outside a Research Ethics Committee approved protocol.*  ***Policy 131****: Procedure new to the NHS – procedure only previously undertaken as part of a clinical trial […] never previously undertaken or used in the NHS*  ***Policy 153****: NICE considers an interventional procedure to be “new” if a fully trained clinician is considering the use of a procedure/technique for the first time in the NHS outside Research Ethics Committee approved protocol.* | 6 |
| Delivery/use is for the first time in the NHS, and it may have been done/used previously in research  ***Policy 023****: A novel procedure is […] one that has only been performed elsewhere under research protocols. It should be a significant development of current practice or arise out of research.*  ***Policy 039****: ‘New’ Interventional Procedure – An interventional procedure should be considered new […] when introducing a procedure as standard practice following a clinical trial*  ***Policy 098****: A new technique and procedure that has been researched within [the trust/health board] and there is good evidence to support adoption*  ***Policy 131****: “New” means any of the following applies: undertaken or used only in context of a research protocol* | 4 |
| **Place *and* Procedure** | **3** |
| Delivery/use is for the first time in the organisation and significantly differs from current practice  ***Policy 023****: A new procedure is one that has not previously been performed within [the trust/health board] or widely elsewhere. It should represent a significant change in patient management. […] or technique that has not previously been used within [the trust/health board]*  ***Policy 118****: The application of existing proven techniques to new indications and not previously carried out at [the trust/health board] […] new to […] to [the trust/health board], which significantly differs from current practice within [the trust/health board],*  ***Policy 156****: Any procedure new to the organisation (even where it has been practiced elsewhere in the NHS), which represents a major change in practice* | 3 |
| **Place *and* External guidance** | **3** |
| Delivery/use is for the first time in the NHS and it is not already registered with NICE IPAC  ***Policy 033****: Types of procedure to be considered by [the committee]: those which are not established in clinical practice within the NHS and have not yet been notified to NICE/IPAC*  ***Policy 108****: not established in clinical practice within the NHS and have not yet been notified to the NICE Interventional Procedures Programme*  ***Policy 156****: Hence, practically a “New Intervention” should more usefully be defined as one of the following: One new to the NHS and not already registered with the NICE IPAC group*. | 3 |
| **Place *and* Economic** | **2** |
| Delivery/use is for the first time in the organisation and there are financial implications  ***Policy 154****: It is a new procedure to the organisation and has implications relating to cost and/or involvement of other services or professions*  ***Policy 156****: Any procedure new to the organisation (even where it has been practiced elsewhere in the NHS), which represents a major change […] significant change in costs.* | 2 |
| **Procedure** | **70** |
| A major modification will be made | 42 |
| … but the policy does not provide a definition of ‘modification’  ***Policy 007****: It is a major modification of an existing procedure*  ***Policy 018****: Policy applies to significant changes to clinical practice or the introduction of new procedures; it does not apply to minor incremental changes or developments.*  ***Policy 021****: Major modifications to clinical techniques and interventional procedures […] it is significantly different from procedures already performed by this clinical team for the same indications*  ***Policy 032****: The Policy applies to significant changes in practice*  ***Policy 033****: This Policy applies to significant changes to clinical practice or the introduction of new procedures; it does not apply to minor incremental changes or developments.*  ***Policy 036****: A significant variation in technique or development of an established procedure.*  ***Policy 049****: or a significant modification to an existing technique or medical device.*  ***Policy 057****: A procedure, medical device or process of clinical management which is substantially different from the alternative*  ***Policy 060****: Exclusions: minor incremental changes or developments*  ***Policy 061****: major modifications of established practice*  ***Policy 063****: is a significant modification of an established treatment or procedure […] A significant modification should be treated like a completely new procedure.*  ***Policy 066****: New techniques including major modifications of current procedures*  ***Policy 067****: New techniques including major modifications of current procedures. […] Major changes in clinical practice or changes to published [the trust/health board] guidelines*  ***Policy 072****: introducing a new/modified procedure […] modified procedure to the organisation. The approval process aims to address [...] the modification of existing techniques*  ***Policy 076****: [the trust/health board] recognises that the majority of therapeutic techniques are generic in nature and subject to variation in their delivery by different clinicians. Variations and modifications of such techniques are commonly required due to variations in clinical presentation. These constitute minor modifications and are not subject to this Policy.*  ***Policy 081****: EXCLUSIONS FROM POLICY: If the technique involves minor incremental changes to or within an established practice*  ***Policy 092****: This Policy applies to significant changes to clinical practice or the introduction of new procedures; it does not apply to minor incremental changes or developments.*  ***Policy 096****: significant modifications to existing procedures […] This Policy does not apply to minor incremental changes to practice, but some changes in current practice may be sufficiently radical as to require approval*  ***Policy 098****: Significant variation or further development of an already established procedure.*  ***Policy 102****: This Policy is focused on significant variation that leads to a new interventional procedure rather than modification of a current procedure. If doubt exists as to whether a procedure should be considered new, this Policy should be followed. […] a significant variation in technique*  ***Policy 104****: do not include minor incremental changes or developments.*  ***Policy 106****: the introduction of new and modified clinical procedures. […] Introduction of new clinical skills by any health care professional*  ***Policy 107****: Any major modifications to an established procedure*  ***Policy 108****: This Policy applies to significant changes to practice or the introduction of new procedures; it does not apply to minor incremental changes or developments.*  ***Policy 109****: any new interventional procedure, which goes beyond minor incremental changes or developments to their clinical practice*  ***Policy 110****: represent a substantial change to existing methods of practice […] The process will not include […] minor incremental changes or developments*  ***Policy 118****: A significant variation in technique,*  ***Policy 119****: This Policy applies to significant changes to practice or the introduction of new procedures; it does not apply to minor incremental changes or developments.*  ***Policy 134****: It is a modification of an established treatment or procedure*  ***Policy 139****: techniques, interventions and medications already used in [the trust/health board] but which are being proposed for use in a different way than is currently used […] introduce a new techniques, equipment, technology and medications which significantly differs from current practice.*  ***Policy 147****: any new procedure, which goes beyond minor incremental changes or developments to their clinical practice,*  ***Policy 148****: it is a modification of an established treatment or procedure […] A major modification should be treated like a completely new procedure. If the original treatment require approval by [the committee], any proposed change will need approval.*  ***Policy 153****: major modifications of established practice,* | 33 |
| … and the policy provides an illustrative example of a technical major modification to an invasive procedure  ***Policy 016****: A move from open surgery to endoscopic procedure would be new to [the trust/health board] and would require the completion of a new procedure proposal.*  ***Policy 053****: A move from open surgery to endoscopic procedure would be new to [the trust/health board] and would require the completion of a new procedure proposal.*  ***Policy 056****: A new hernia mesh that works in a different way would not be a minor modification and should follow [this Policy]*  ***Policy 083****: Incremental improvements to existing practice due to changes in technique proposed by professional bodies are not considered a new procedure […] A move from open surgery to endoscopic procedure would be new to [the trust/health board] and would require the completion of a new procedure proposal.*  ***Policy 084****: Incremental improvements to existing practice due to changes in technique proposed by professional bodies are not considered a new procedure. […] A move from open surgery to endoscopic procedure would be new to [the trust/health board] and would require the completion of a new procedure proposal*  ***Policy 091****: A move from open surgery to endoscopic procedure would be new to [the trust/health board] and would require completion of a new procedure proposal.*  ***Policy 123****: A move from open surgery to endoscopic procedure would be new to [the trust/health board] and would require completion of a new procedure proposal.*  ***Policy 125****: which significantly differs from current clinical practice […] A move from open surgery to endoscopic procedure would be new to [the trust/health board] and would require completion of a new procedure proposal.*  ***Policy 131****: A key question is – what is a new procedure? Sometimes this is clear, the move from open to laparoscopic surgery was a clear change, replacing a joint where previously repair or simply removal was undertaken is new. […] Documenting the change at the local governance group as a minor adaptation but not new procedure is a helpful safeguard* | 9 |
| **Delivery/use is in a different clinical circumstance, i.e.** | **24** |
| … for a new indication  ***Policy 118****: The application of existing proven techniques to new indications*  ***Policy 003****: Extension of a currently used interventional procedure to a new clinical circumstance should also be considered a new procedure.*  ***Policy 007****: a transfer of a technique, procedure or method into a different clinical field or diagnosis*  ***Policy 010****: A treatment modality and equipment already in use in [the trust/health board] but to be used for a different purpose by a practitioner as an extension to their practice.*  ***Policy 012****: New procedures may fall into one of the following three categories: The application of existing proven techniques to new indications.*  ***Policy 022****: New procedures may fall into one of two categories: The application of existing proven techniques to new indications/conditions;*  ***Policy 027****: Will the new technology be used with the same patient group? Will the new device be used for a new clinical indication?*  ***Policy 036****: An established procedure extended to a new condition.*  ***Policy 039****: Include the application of an existing technique to a new clinical purpose*.  ***Policy 043****: A treatment modality and equipment already in use in the Trust but to be used for a different purpose by a practitioner as an extension to their practice.*  ***Policy 054****: The application of an existing proven procedure(s) to new indications.*  ***Policy 055****: an existing interventional procedure being applied […] for a different indication. […] new indication for an existing interventional procedure*  ***Policy 088****: New Interventional Procedure - an existing procedure […] for a different indication. […] New […] indication of existing interventional procedure […] Use of an existing procedure but for a new indication*  ***Policy 094****: An existing treatment/technique that is to be adopted/adapted for a new purpose.*  ***Policy 098****: An established procedure extended to a new condition.*  ***Policy 102****: The application of existing proven techniques to new indication*  ***Policy 116****: a new combination of existing technologies/procedures currently in use in [the trust/health board]. […] Will the variation be used in the same patient population? Will the variation be used for a new clinical indication? Does the variation represent a change in clinical practice?*  ***Policy 138****: The application of existing proven techniques to new indications*  ***Policy 139****: Is this an existing technique, intervention and/or medication but being proposed for a different purpose/use with a different patient group? Is this a technique, intervention and/or medication that is being used effectively in the treatment of adults, but only starting to be used in paediatrics?*  ***Policy 156****: The extension of a procedure currently used within the organisation to a new cohort of patients* | 20 |
| … in a different part of the body  ***Policy 001****: If there is any uncertainty as to whether the proposed intervention classifies as a ‘new intervention’ (e.g. where the intended procedure may be a modification of an existing one, e.g. a familiar technique but a different access site)*  ***Policy 025****: An established procedure extended to a new condition or part of the body.*  ***Policy 055****: an existing interventional procedure being applied to a different part of the body […] new site […] for an existing interventional procedure*  ***Policy 088****: New Interventional Procedure - an existing procedure being applied to a different part of the body […] New site […] of existing interventional procedure Approval will only cover use of the IP for the site on the body at which the original application was made. Use at a new site must first be approved by [the committee].*  ***Policy 131****: However, there are many less obvious adaptations – using an established technique for a different site (cryotherapy for example) or increasing the scope or extent of a laparoscopic technique.* | 5 |
| … within a new combination of treatments  ***Policy 012****: These may be combined in such ways that a new device or technique opens the way for the management of a condition perhaps not previously treated, or managed in a totally different way*  ***Policy 116****: a new combination of existing technologies/procedures currently in use in [the trust/health board].*  ***Policy 130****: new combinations of existing treatments*. | 3 |
| Delivery of the procedure involves the use of new devices or equipment  ***Policy 010****: A technique where new instrumentation has to be used including a new purchase or loan equipment either requiring or not requiring consumables.*  ***Policy 012****: A significant variation in technique, new equipment or devices […] used during the course of a standard procedure,*  ***Policy 016****: New Clinical Procedure: any clinical intervention which involves new techniques which have not previously been undertaken by [the trust/health board]; it may also include the use of new equipment.*  ***Policy 023****: If a procedure is not new or novel but involves the use of a new device or technique that has not previously been used within [the trust/health board] […] [the committee] will also examine the introduction of devices or materials developed and used in the USA for which there is no current UK or European license.*  ***Policy 026****: New Clinical Procedure: any clinical intervention which involves new techniques which have not previously been undertaken by [the trust/health board]; it may also include the use of new equipment.*  ***Policy 039****: A new use for existing equipment.*  ***Policy 043****: A technique where new instrumentation has to be used including a new purchase or loan equipment either requiring or not requiring consumables*  ***Policy 052****: It may be difficult to differentiate between a modification of a current procedure and a new procedure. Changes in suture material would probably be a modification (provided the suture material itself was not new) whilst the use of a new device to facilitate surgery would be a new procedure*  ***Policy 053****: New Clinical Procedure: any clinical intervention which involves new techniques which have not previously been undertaken by [the trust/health board]; it may also include the use of new equipment,*  ***Policy 058****: new techniques for clinical practice - which might include the use of new devices - introduced to [the trust/health board].*  ***Policy 065****: all techniques or procedures (including the use of novel equipment) that are new to [the trust/health board]*  ***Policy 066****: New devices or technologies.*  ***Policy 067****: For this purpose, in [the trust/health board], new procedures include […] New devices e.g. Prosthesis.*  ***Policy 083****: New Clinical Procedure: any clinical intervention which involves new techniques which have not previously been undertaken by [the trust/health board]; it may also include the use of new equipment.*  ***Policy 084****: New Clinical Procedure: any clinical intervention which involves new techniques which have not previously been undertaken by [the trust/health board]; it may also include the use of new equipment.*  ***Policy 101****: ‘A new procedure is difficult to define strictly but would include: one for which there is a major modification in technique that requires new equipment*  ***Policy 102****: the application of new equipment/device during the course of a standard procedure.*  ***Policy 113****: For a new implantable medical device*  ***Policy 118****: new equipment or new device used during the course of a standard procedure*  ***Policy 123****: New Clinical Procedure: any clinical intervention which involves new techniques which have not previously been undertaken by [the trust/health board]; it may also include the use of new equipment.*  ***Policy 131****: The Policy covers […] new medical devices (including protheses) being introduced either into an established or new procedure.*  ***Policy 136****: Surgical equipment and the use of equipment new to the surgeon him/herself.*  ***Policy 140****: New Clinical Procedure: any clinical intervention which involves new techniques which have not previously been undertaken by [the trust/health board]; it may also include the use of new equipment*. | 23 |
| Delivery/use requires enhanced/modified consent  ***Policy 025****: “New” means completely novel or a sufficient adaptation of a technique that makes it different in such a way that enhanced or modified patient consent is required […] A key test is to consider what you would say if an incident or complication occurred and you were subsequently being open.*  ***Policy 055****: As a guide, a significant modification is one whereby the information given to the patient during the consent process requires amending*  ***Policy 064****: Does the introduction of this new technology generate additional risk beyond routine consent?*  ***Policy 088****: As a guide, a significant modification is one whereby the information given to the patient during the consent process requires amending* | 4 |
| A minor modification will be made, but the policy does not provide a definition of ‘modification’  ***Policy 116****: All minor amendment to and existing clinical technology or procedure must be introduced in line with processes described within this Policy [..] Minor amendment refers to a change in the way in which an existing technology/procedure is undertaken (including modifications to existing devices) and which does not require users to receive additional training or proctorship.*  ***Policy 121****: A technique is classed as new if a minor change is made to a procedure which is already performed* | 2 |
| Different aftercare is required  ***Policy 131****: the changed intervention requires different aftercare;* | 1 |
| **Evidence** | **42** |
| There are uncertain or changed outcomes  ***Policy 002****: ‘New’ must be judged according to whether the altered technique is likely to have a different safety and efficacy profile from that of current alternatives. […] This Policy does not relate to changes in practice that can be considered to be minor incremental changes or developments […] A judgement may be required as to whether or not a procedure has been altered in some minor incremental way, or whether sufficient alteration has been made for it to constitute a new procedure*  ***Policy 004****: A judgement may be required as to whether or not the technique has been altered in some minor incremental way, or whether sufficient alteration has been made for it to constitute a new technique. This should be judged according to whether the altered technique is likely to have a different safety and efficacy profile from that of the current alternatives. […] Performing an established procedure using a* *new device would not usually fall within the remit of the programme unless the use of the new device appeared to alter the safety and efficacy profile of the procedure.*  ***Policy 012****: A significant variation in technique, new equipment or devices […]used during the course of a standard procedure, i.e. whether the new technique is likely to have a different safety and efficacy profile from that of the original procedure.*  ***Policy 013****: This includes; modifications of existing techniques where there are additional or new potential clinical risks*  ***Policy 019****: Further, a ‘new’ procedure represents a substantial change in practice in terms of: risk to the patient, practitioner and organisation*  ***Policy 022****: A significant variation in technique, new equipment or new device used during the course of a standard procedure i.e., whether the new technique is likely to have a different safety and efficacy profile from that of the original procedure. [NB A significant modification to an existing technique could also be deemed a new procedure].*  ***Policy 033****: those for which there are uncertainties about their safety and/or efficacy as specified in the guidance issued by NICE/IPAC,*  ***Policy 038****: Where the new procedure is a minor modification of an existing procedure which has little impact on safety or efficacy, the clinical director may judge that suitable arrangements are already in place and further approval is not required.*  ***Policy 039****: Including procedures, interventions or techniques which: Are substantially different from any procedure currently in place, or Are a major modification (technical or conceptual) of an existing procedure, likely to result in changed outcomes in terms of efficacy or safety*  ***Policy 041****: For the purpose of this Policy significant change or new procedure which is deemed to be “any clinical change that has an impact on either the patient’s experience or outcome”*  ***Policy 051****: ‘First Time’ Procedures: They are entirely novel, with an unknown or uncertain efficacy and/or safety profile; or they are a variation of an established procedure which is likely to have a different efficacy and/or safety profile from that of the established procedure. […] This may be in the opinion of the healthcare professional, specialty business unit Manager/Associate Director or Departmental/general Manager, being of significant clinical risk […] When a staff member wants to undertake a technique intervention procedure or use equipment for the first time, and could be considered significant risk to the patient/[the trust/health board] if not performed in the correct manner.*  ***Policy 054****: Performing an established procedure using a new device would not usually fall within the remit of this Policy unless the use of the new device appeared to alter the safety and efficacy profile of the procedure itself.*  ***Policy 057****: New Interventional Procedure, Technology or Treatment: A procedure, medical device or process of clinical management which is substantially different from the alternative, and which has the potential to adversely impact on patient safety, efficacy*  ***Policy 058****: An interventional procedure should be considered New […] when an existing procedure is significantly modified in terms of management and risk. […] This procedure does not cover minor modifications of existing procedures where safety and efficacy are not in question*  ***Policy 061****: Performing an established procedure using a new device would not usually fall within the remit of the Policy unless the use of the new device appears to alter the safety and efficacy of the procedure.*  ***Policy 065****: However if the new procedure or equipment could reasonably be regarded as no more than a minor variation of a current procedure or instrument already in use in the trust, and in particular carries no additional risk […] then consideration by [the committee may not be needed];*  ***Policy 076****: However this Policy also applies to: The intention to introduce a planned Major modification or substantial degree of difference (technical or conceptual) of an existing procedure which could result in changed outcomes in terms of efficacy or safety.*  ***Policy 092****: Those for which there are uncertainties about their safety and/or efficacy as specified in guidance issued by NICE-IPAC*  ***Policy 101****: In general, minor modifications of existing procedures where safety and efficacy are not in question would not be included.*  ***Policy 108****: Those for which there are uncertainties about safety and/or efficacy as specified in Interventional Procedures Programme process guide issued by NICE*  ***Policy 118****: Either not yet generally considered standard clinical practice or Be standard clinical procedure, the safety and efficacy of which has been called into question by new information*  ***Policy 119****: If a clinician wishes to introduce a new procedure or technique which is not considered standard practice, or where the risks and benefits are uncertain, advice should be sought from the National Institute of Clinical Excellence (NICE).*  ***Policy 126****: A novel procedure associated with uncertainty about its safety or efficacy.*  ***Policy 131****: Consideration needs to be given to whether: risks change in changing the intervention*  ***Policy 137****: Clinicians are responsible for ensuring that changes made to an already provided procedure, which may have a sizeable impact on the benefit or risk profile of that procedure or are outside established practice, are discussed with clinical and managerial colleagues.*  ***Policy 138****: A significant variation in technique, new equipment or new device used during the course of a standard procedure, i.e. whether the new technique is likely to have a different safety and efficacy profile from that of the original procedure.*  ***Policy 153****: In practice this Policy applies to any circumstance in which any healthcare professional proposes to carry out a clinical procedure with which they are unfamiliar and which, by its nature, may expose the patient to risk […] Performing an established procedure using a new device would not usually fall within the remit of the Policy unless the use of the new device appears to alter the safety and efficacy of the procedure.*  ***Policy 154****: It involves a major modification (technical or conceptual) of an existing procedure, likely to result in changed outcomes in terms of efficacy or safety c) It involves the application of an established technique to a new clinical purpose and is likely to result in changed outcomes in terms of efficacy and safety d) It has limited previous use in the UK and is likely to result in changed outcomes in terms of efficacy and safety […] g) It has a potential for significant risk h) There is limited published evidence regarding its safety and efficacy* | 28 |
| Currently being delivered within a research study | 17 |
| … within or outside of the organisation  ***Policy 013****: A procedure is to be undertaken for the first time as part of a research project.*  ***Policy 032****: Techniques and procedures that form an accepted part of an approved research programme.*  ***Policy 091****: If NICE has indicated that the procedure is within its category ‘Research Only’ (or ‘Other’ category recommending the procedure should only be undertaken as part of research) the health professional should initially gain approval from [the trust/health board] [Research and Development unit], and Local Research Ethics Committee, before following this Policy and submitting an application to [the committee].*  ***Policy 101****: ‘A new procedure is difficult to define strictly but would include: one that is part of a research project;*  ***Policy 113****: A research study for this application is a project which has been devised to establish whether a new procedure or new IMD is effective. It requires appropriate research, ethics and financial approvals before application to [the committee].*  ***Policy 137****: No patients should undergo a new procedure that was first introduced in [the trust/health board] through research without specific authorisation from [the committee].*  ***Policy 146****: This also applies to any new high risk interventional procedure which is performed as part of a trial, including those which have been approved by the [Research and Development unit].* | 7 |
| … and it will be delivered/used outside of the research protocol  ***Policy 012****: Use of the procedure outside the clinical trial protocol must only occur after approval from [the committee] as set out above*  ***Policy 013****: Use outside the protocol should only occur after approval by the process outlined in this Policy*  ***Policy 018****: Use outside the protocol should only occur after approval from [the committee] as set out above.*  ***Policy 036****: Use outside of the protocol should only occur after approval from the Medical Director*  ***Policy 071****: Use outside of the protocol should only occur after approval from [the committee].*  ***Policy 076****: Use outside the protocol should only occur after approval from [the committee] as set out in this document.*  ***Policy 081****: Use outside the protocol should only occur after approval from [the committee] as set out above.*  ***Policy 104****: The procedure however cannot be used outside the [research] protocol unless approval/ ratification has been obtained from [the committee] […] as outlined in this Policy.*  ***Policy 122****: Use outside the protocol should only occur after approval from [the committee].*  ***Policy 133****: Use outside the protocol should only occur after approval from [the committee].*  ***Policy 137****: Where [the committee] has authorised a NIP as part of a research protocol, further authorisation must be sought to undertake the procedure outside of the protocol/once the research study has completed.*  ***Policy 138****: Use outside the protocol must only occur after approval from [the committee] as set out above.*  ***Policy 146****: Use outside the protocol should only occur after approval from [the committee] as set out above.* | 13 |
| Delivery/use is prior to the commencement of a research study  ***Policy 055****: This Policy applies to all clinical staff undertaking interventional procedures across [the trust/health board] and covers: [...] pre-trial new interventional procedures* | 1 |
| Delivery/use has previously been halted at the trust  ***Policy 042****: New procedures include those […] which were performed in the past but use of them was ceased at [the trust/health board].* | 1 |
| Delivery/use is to demonstrate proof of concept  ***Policy 116****: Experimental medicine refers to any new technology/procedure being introduced to ‘demonstrate proof-of-concept evidence for the validity and importance of new discoveries or treatments’ (www.mrc.ac.uk )* | 1 |
| **Economic** | **10** |
| Delivery/use has financial/resource implications  ***Policy 003****: New practice that has resource implications resulting in a request for additional funds from commissioners […] New practice that might have impacts (clinical governance or resource) beyond a single clinical directorate […] If the new clinical practice impacts on another directorate or it requires additional resource to implement*  ***Policy 010****: A Modification to an interventional procedure is defined as one that could have […] impacts on resource cost*  ***Policy 019****: Further, a ‘new’ procedure represents a substantial change in practice in terms of: […] resource implications in terms of nursing and medical staff, medical equipment, in-patient stay, etc.*  ***Policy 041****: For the purpose of this Policy significant change or new procedure which is deemed to be “any clinical change that has an impact on […] financial resources”.*  ***Policy 043****: A modification to an interventional procedure is defined as one that […] impacts on resource cost*  ***Policy 057****: New Interventional Procedure, Technology or Treatment: A procedure, medical device or process of clinical management which is substantially different from the alternative, and which has the potential to adversely impact […] cost effectiveness.*  ***Policy 080****: Where a change in practice (minor or major in clinical terms) would have financial consequences*  ***Policy 082****: Minor changes to techniques that are consistent with 'accepted clinical practice' (and are already well established in other NHS [trusts/health boards]) and which do not represent a significant departure from local practice in terms of […] resources required for their local implementation do not need to be evaluated fully as above.*  ***Policy 115****: The Policy does not apply to minor incremental changes to existing procedures unless there is a significant funding […] issues requiring [the committee] approval.*  ***Policy 136****: For the purposes of this Policy. 'significant change' is deemed to be 'any change that has an impact on […] resources including financial resource'* | 10 |
| **External guidance** | **3** |
| Recommendations from NICE or other national/international bodies  ***Policy 061****: All new […] procedures with NICE Guidance must be agreed before introduction*  ***Policy 098****: A NICE Interventional Procedure that has been approved for use and is no longer within the research specification.*  ***Policy 126****: new IPs which have been recommended by NICE,* | 3 |

^1^Policies may be coded to more than one theme

NHS = National Health Service; NICE = National Institute for Health and Care Excellence; IPAC = Interventional Procedures Advisory Committee; REC = Research Ethics Committee
