# Supplementary material for: Healthcare organization policy recommendations for the governance of surgical innovation: review of NHS policies
Source: Br J Surg. 2022 Jul 30;109(10):1004–12. doi: 10.1093/bjs/znac223 (PMC10364689; doi:10.1093/bjs/znac223)
Supplement: znac223_Supplementary_Data [file znac223_supplementary_data.zip › Supplementary Table 2_Final.docx]

Supplementary Table 2. Verbatim policy text coded to themes describing when new invasive procedures and devices are recommended for research ethics committee application

| **Over-arching theme**  Individual theme  Sub-theme  *Verbatim policy text* | **Number of policies with text coded to theme, n=34^1^** |
| --- | --- |
| **External Guidance** | **18** |
| Classified by NICE/NICE IPAC as to be delivered within research  ***Policy*** ***001****: When NICE classify the arrangement type of a particular procedures as Research only (use only in the context of a research protocol), the default position of [the committee] will be to refuse the application, and to redirect the applicant to considering the procedure within the context of a research study.*  ***Policy*** ***010****: Where practitioners have expressed an interest in performing a new interventional procedure, the following actions will be taken according to the NICE classification: Research Only – practitioners will be referred to the Research Office.*  ***Policy*** ***033****: Procedures introduced as part of a research project: If there is uncertainty about a procedure and/or NICE-IPAC guidance advises it, they must only be approved for a Healthcare Professional or use within [the trust/health board] and undertaken as part of an ethically approved formal research study.*  ***Policy*** ***037****: When NICE determine that the evidence on safety and efficacy ‘does not appear adequate to support the routine use of this procedure. It is suitable for use only within good-quality research studies approved by a research ethics committee and with explicit patient consent’, the default position of [the committee] will be to refuse the application, and to redirect the applicant to considering the procedure within the context of a research study.*  ***Policy*** ***043****:[Flow diagram] NICE classification = research only. Applicant is directed to the [research office].*  ***Policy*** ***055****: NICE assessment of a new interventional procedure may conclude that there is inadequate evidence on its safety and efficacy and that it must therefore only be undertaken in the context of clinical research. This does not mean that a research application is automatically approved […] The Chair of the Research Ethics Committee should advise whether the procedure falls into the research category.*  ***Policy 059:*** *Furthermore if NICE have advised that the procedure only be carried out as part of a clinical research trial the proposing clinician will be referred to the Research and Development Department*  ***Policy*** ***063****: if NICE requires to be done as part of research trial or with special monitoring either: Submit to [the trust/health board] Research Ethics Committee AND [the committee]; If not part of research, submit to [the committee].*  ***Policy 066****: NICE status - please tick: see* [*www.nice.org.uk/ip*](http://www.nice.org.uk/ip) *[...] B: Part of an approved research programme – requires approval by the R&D Department and LREC*  ***Policy 067:*** *NICE status - please tick: see* [*www.nice.org.uk/guidance*](http://www.nice.org.uk/guidance) *[...] B: Part of an approved research programme – requires approval by the R&D Dept and HRA (please visit [organisation website])*  ***Policy 088:*** *NICE assessment of a new IP may conclude that there is inadequate evidence on its safety and efficacy and that it must therefore only be undertaken in the context of clinical research. This does not mean that a research application is automatically approved; the standard R&D approval processes must be followed. Therefore, new IPs which NICE recommend may only be undertaken in the context of research may not be introduced into routine clinical practice until the NICE recommendation changes, and will not be considered by [the committee].*  ***Policy*** ***091****: Any new procedures undertaken as part of research should be considered first through [the trust/health board] research policy and processes (included where indicated within NICE’s category of ‘Research Only’). […] If NICE has indicated that the procedure is within its category ‘Research Only’ (or ‘Other’ category recommending the procedure should only be undertaken as part of research) the health professional should initially gain approval from [the trust/health board] [research and development unit] and Local Research Ethics Committee, before following this policy and submitting an application to [the committee].*  ***Policy*** ***092****: If […] NICE-IPAC guidance advises it, they must only be approved for use within [the trust/health board] and undertaken as part of an ethically approved formal research study.*  ***Policy*** ***108****: Procedures Introduced as part of a Research Project: The procedure should be undertaken as part of a formal research study if either: […] it has been recommended by the NICE Interventional Procedures Programme.*  ***Policy 154:*** *7.1.6 If the procedure described in the NICE Interventional Procedure Guidance is undertaken within the organisation, but the guidance indicates that the procedure must only be undertaken as part of an ethically approved research study, then either: Its use must cease until a research application has been made and has been approved by the [trust/healthboard] R&D Department and the appropriate Research Ethics Committee (refer to [organisation document]). The healthcare professional and the Service Delivery Team, Directorate Management Team and the R&D Department will be informed of this requirement in writing by the Clinical Effectiveness Co-ordinator so that they can take appropriate action. Or If there is no intention to participate in a research study then its use must cease immediately.* | 15 |
| There is no NICE or national best practice guidance  ***Policy*** ***034****: If no NICE guidance: [Research and Development Unit] critically appraises the evidence or identifies opportunities for participation in multi-centre trials.*  ***Policy*** ***068****: For cases where national best practice is not available, a submission to the [Research and Development unit] will be required. If deemed inappropriate for Research approval then a further review will be undertaken by […] [the committee].*  ***Policy*** ***128****: All new techniques not covered by NICE guidance will be scrutinised and a decision made as to whether they would best be dealt with by a research approach and as such the clinician(s) may be asked to progress the technique through the [research and development unit].* | 3 |
| **Evidence** | **13** |
| There is an insufficient evidence base to support delivery or use of the invasive procedure or device:  … but no definition of insufficient/sufficient is given.  ***Policy*** ***082****: Where the innovation is a treatment […] and the required standard of evidence is not met, then the technique must not be introduced to [the trust/ health board]. Rather, consideration should be given to the local introduction of the innovation, not as new clinical practice but as 'experimental clinical practice' within the context of a local research study, usually a controlled clinical trial. Such cases should be discussed with […] and referred to the [research and development unit].*  ***Policy*** ***092****: Is the procedure a new or incremental improvement? Is the evidence base for the procedure confirmed? If no: Will not be considered. Instead consider enrolling in national trial / research study; Direct to [Research and Development unit]*  ***Policy*** ***114****: Where an adequate evidence base does not exist, then novel treatments may still be introduced […] in discussion with the medical director, but in a way so that the experience gained can form part of an ongoing study, part of an original study or contribute in some other way to the written and published experience with the proposed treatment.*  ***Policy*** ***140****: The National Health Service (NHS) and [the trust/health board] need to ensure that: All new procedures and techniques introduced are evidence based or they will form part: of a Research Ethics Group (REC) and [research and development unit] approved trial […] This policy does not apply to: Any procedure which is part of a research study when the governance procedures would apply; […] Procedures introduced as part of a research project: Where a clinician wishes to introduce a new procedure and either s/he or [the committee] takes the view that evidence for its safety and/or effectiveness and/or cost-effectiveness is inadequate, the procedure may only be introduced in the context of an appropriately designed research study.* | 6  4 |
| There is an insufficient evidence base to support delivery or use of the invasive procedure or device:  …and the required level of evidence is specified  ***Policy*** ***001****: Is the procedure in use elsewhere, as described in a peer-reviewed publication? If no, the this looks as [if] this is a brand new procedure without an existing supportive evidence-base, and could be evaluated within the context of a formal research study. Please contact head of [research and development unit] for further advice before proceeding further.*  ***Policy*** ***068****: Is there a sufficient body of evidence supporting the use of this practice nationally or internationally in recognised centres? Have you submitted request to [Research & Development unit]? Submit request to [research and development unit].* | 2 |
| There are uncertain outcomes:  … related to safety/efficacy  ***Policy*** ***015****: SERNIP Definition: Safety and/or efficacy not yet established; procedure required a fully controlled evaluation and may be used only as part of systematic research, comprising of an observational study in which all interventions and their outcomes are systematically recorded - Requires application through [research and development unit]. Translation into routine practice would require [the committee] scrutiny*  ***Policy*** ***082****: Where the innovation is a treatment or procedure that could affect patient safety and clinical outcome […] then the technique must not be introduced to the Trust. Rather, consideration should be given to the local introduction of the innovation, not as new clinical practice but as 'experimental clinical practice' within the context of a local research study, usually a controlled clinical trial. Such cases should be discussed with the Chief of Service and / or Director of Clinical Service and referred to the Research & Innovation Department.*  ***Policy*** ***140****: Procedures introduced as part of a research project: Where a clinician wishes to introduce a new procedure and either s/he or [the committee] takes the view that evidence for its safety and/or effectiveness and/or cost-effectiveness is inadequate, the procedure may only be introduced in the context of an appropriately designed research study.* | 5  3 |
| There is any uncertainty related to delivery or use  ***Policy*** ***092****: If there is uncertainty about a procedure […] they must only be approved for use within [the trust/health board] undertaken as part of an ethically approved formal research study.*  ***Policy*** ***108****: Procedures Introduced as part of a Research Project: The procedure should be undertaken as part of a formal research study if either: there is uncertainty about it […]* | 2 |
| The invasive procedure/device is ‘original’, ‘completely new’ or ‘experimental’ (no definitions given)  ***Policy*** ***044****: Has the procedure been carried out elsewhere? If Yes, is it: Experimental? Routine Practice? NB If the procedure is experimental it should be treated as an R & D proposal*  ***Policy*** ***059****: Has this procedure been carried out elsewhere? If yes, is it Experimental or Routine Practice (NB if experimental it should be treated as an R&D proposal)*  ***Policy*** ***076****: Therefore in the case of original interventional procedures, these must be considered as a research study and [the trust/health board] Policy on Research Governance must be followed.*  ***Policy*** ***081****: An explanation must be given to the patient as to whether the new technique / procedure is either new to [the trust/health board] but established elsewhere, or, is a completely new technique / procedure. In the latter circumstance such a completely new technique / procedure must have had full ethical and research approval and documentation in accordance with [the trust/health board] and national requirements.* | 4 |
| Delivery/use is within a project to determine its effectiveness  ***Policy*** ***113****: A research study for this application is a project which has been devised to establish whether a new procedure or new IMD is effective. It requires appropriate research, ethics and financial approvals before application to [the committee].* | 1 |
| **Evidence *and* External guidance**  The invasive procedure/device is not established in clinical practice and delivery/use has not been notified to NICE IPAC  ***Policy 033:*** *This policy is concerned with three types of procedures: […] Those which are not established in clinical practice within the NHS and have not yet been notified to NICE-IPAC. These procedures should be referred for consideration as part of a national trial or research*  ***Policy 092****: Those which are not established in clinical practice within the NHS and have not yet been notified to NICE-IPAC. These procedures should be referred for consideration as part of a national trial or research. […] Procedures introduced as part of a research project: If there is uncertainty about a procedure and/or NICE-IPAC guidance advises it, they must only be approved for use within [the trust/health board] and undertaken as part of an ethically approved formal research study.* | **2**  2 |
| **Place** | **7** |
| Delivery/use is for the first time anywhere  ***Policy*** ***019****: This following protocol must be followed if: The procedure is entirely innovative and has not been performed elsewhere. It is not expected that such new procedures in this category will be undertaken in [the trust/health board]. Of necessity this would form part of a full programme of Clinical Research and Development overseen by Research Ethics Committee or if being introduced into clinical practice requires adherence to Department of Health’s Circular HSC 2003/011.*  ***Policy*** ***025****: New procedures can be divided into the following categories: The procedure has never before been undertaken anywhere. In this case the proposal is research and should follow standard research governance protocols, registration with [the research and development unit] and will include a submission to the relevant Research Ethics Committee. This policy does not apply to these procedures.*  ***Policy*** ***036****: New procedures can be divided into the following categories: The procedure has never before been undertaken anywhere. (In this case the proposal is research and should follow standard research governance protocols, registration with [the research and development unit] and will include a submission to the relevant Research Ethics Committee).*  ***Policy*** ***050****: Entirely new - never performed anywhere else? Has ethical approval been obtained from Research Ethics Committee (REC)? If Yes, provide reference. […] The proposal should include the following information: An indication as to whether it is an entirely new procedure never performed anywhere else and if so whether ethical approval has been obtained, or whether it is a procedure that is new to [the trust/health board] but has been performed elsewhere.*  ***Policy*** ***054****: A procedure that has never been tried before. This will need to be passed by the Local Research Ethics Committee (REC) and evaluation should follow standard research protocols*  ***Policy*** ***082****: There is a range of circumstances that may be considered a “new technique”. This would range from the development of a brand new procedure that had never been undertaken in humans (i.e. original research) […] Truly novel clinical innovations, i.e. 'inventions' of new clinical procedures, must be referred to [the research and development unit]. Their introduction must only occur within the context of a well designed research study that has been approved by a research ethics committee and has received local research governance and management approval. […] Is this newly invented Y/N? If yes - discuss with [the research and development unit] re Clinical Trial*  ***Policy*** ***098****: Excluded areas: A new procedure never undertaken anywhere before. If this is the case the proposal will be considered as research and should follow the normal pathway for research proposals* | 7 |
| **Place *and* Evidence**  The invasive procedure/device has not previously been performed in the organisation and is not established in clinical practice  ***Policy*** ***049****: If it has not previously been used in [the trust/health board] and is not an established technique and/or device: It is the responsibility of applicants to [the committee] to ensure that they have sought appropriate independent review of their proposal and to establish whether an application to a Research Ethics Committee is required.* | **1**  **1** |
| **Personnel** | **2** |
| Delivery/use requires additional training  ***Policy*** ***056****: New Interventional Procedures/Devices which are entirely new to the National Health Service: If the profession considers a procedure or treatment sufficiently novel as to require special training and assessment before being introduced into clinical practice, then its use should be limited to a number of specified centres. Healthcare professionals at [the trust/health board] may wish to be involved in these clinical trials. These should be in accordance with [the research and development unit] procedures and follow the Research Governance protocols.*  ***Policy*** ***106****: Entirely new and innovative procedures: If the profession considers a procedure or treatment is sufficiently novel as to require special training and assessment before being introduced into clinical practice, then its use should be limited to a number of specified centres for clinical trials. Health care professionals are encouraged to participate in clinical trials. During the clinical trials, methods of training and training requirements should be developed and identified and the Local Research Ethics Committee (L.R.E.C) and [the research and development unit] co-ordinator should be involved.* | 2 |
| **Procedure** | **1** |
| The invasive procedure involves gene therapy  ***Policy*** ***041****: Is Research Ethics Committee Opinion Required? Based on existing guidance, procedure carried out on request and specifically tailored for the patient, which does not involve xenotransplantation, and is not carried out in the context of research, but as part of clinical treatment can be considered as innovative therapy and as such does not require ethical approval. This is further supported by the report originally provided by the Gene Therapy Advisory Committee (as incorporated in the Health Research Authority – HRA in 2012). Innovative treatment is only considered research if gene therapy is included. Based on the relevant information treatment, which does not form part of a clinical trial/study and is purely administered on the request of the patient and as such qualifies as Innovative Treatment and does not require favourable ethical opinion as required by Governance Arrangements for Research Ethics Committees, 2011 (GAfREC).* | 1 |
| ^1^Policies may be coded to more than one theme  NHS = National Health Service; NICE = National Institute for Health and Care Excellence; IPAC = Interventional Procedures Advisory Committee; REC = Research Ethics Committee | |
