# Supplementary material for: Healthcare organization policy recommendations for the governance of surgical innovation: review of NHS policies
Source: Br J Surg. 2022 Jul 30;109(10):1004–12. doi: 10.1093/bjs/znac223 (PMC10364689; doi:10.1093/bjs/znac223)
Supplement: znac223_Supplementary_Data [file znac223_supplementary_data.zip › Supplementary Table 3_Final.docx]

Supplementary Table 3. Over-arching and combined over-arching themes, individual themes and sub-themes describing when new invasive procedures and devices are within local NHS policy remit

| **Over-arching and combined over-arching themes**  Individual theme  Sub-theme | **Number of policies with text coded to theme, n=109^1^** |
| --- | --- |
| **Personnel** | **81** |
| When the individual clinician will deliver/use the invasive procedure/device for the ‘first time’: | 67 |
| … anywhere, and the policy does not state whether it is already in use elsewhere | 31 |
| … anywhere, and the procedure/device may be in use by other clinicians in the NHS or the organisation | 9 |
| … in the organisation, and the ‘competent training clinician’ is not present | 1 |
| … in their NHS clinical practice, but the clinician may have done one or used it outside of the NHS | 45 |
| … in the organisation, although the clinician may have done one or used it outside of the organisation | 10 |
| Delivery/use requires additional training | 26 |
| … Additional training is required | 22 |
| … A proctor is required | 6 |
| Being delivered/used by a different or extended clinical role | 18 |
| Developed personally by a clinician or for an individual patient/case  When any senior clinician involved in the procedure will deliver it for the first time | 6  1 |
| **Personnel *and* Evidence** | **5** |
| When the individual clinician will deliver/use the invasive procedure/device for the ‘first time’ anywhere, and the procedure/device is established | 5 |
| **Personnel *and* Place** | **3** |
| Delivery/use is for the first time by a clinician and for the first time in the organisation | 3 |
| **Place** | **78** |
| Delivery/use is for the first time in the organisation | 69 |
| … but the policy does not state whether it has been done/used or evaluated elsewhere | 44 |
| … and it may have been delivered/used elsewhere | 25 |
| Delivery/use is for the first time in the NHS | 7 |
| … but the policy does not state whether it has been delivered/used outside the NHS | 5 |
| … and it may have been delivered/used previously outside of the NHS | 2 |
| Delivery/use is for the first time anywhere | 6 |
| Delivery/use is for the first time in the division, but may be in use elsewhere in the organisation | 2 |
| Delivery/use is for the first time in the United Kingdom | 2 |
| **Place *and* Evidence** | **31** |
| Delivery/use is for the first time in the organisation, and it has an evidence base, has been evaluated or undergone clinical trials  Delivery/use is for the first time in the organisation, and it is established in clinical practice elsewhere  Delivery/use is for the first time in clinical practice, and it has been delivered/used in a research setting  Delivery/use is for the first time in the NHS, and it may have been done/used previously in research | 13  10  6  4 |
| **Place *and* Procedure** | **3** |
| Delivery/use is for the first time in the organisation and significantly differs from current practice | 3 |
| **Place *and* External guidance**  Delivery/use is for the first time in the NHS and it is not already registered with NICE IPAC | **3**  3 |
| **Place *and* Economic** | **2** |
| Delivery/use is for the first time in the organisation and there are financial implications | 2 |
| **Procedure** | **70** |
| A major modification will be made | 42 |
| … but the policy does not provide a definition of ‘modification’ | 33 |
| … and the policy provides an illustrative example of a technical major modification to an invasive procedure | 9 |
| Delivery/use is in a different clinical circumstance, i.e. | 24 |
| … for a new indication | 20 |
| … in a different part of the body | 5 |
| … within a new combination of treatments | 3 |
| Delivery of the procedure involves the use of new devices or equipment | 23 |
| Delivery/use requires enhanced/modified consent | 4 |
| A minor modification will be made, but the policy does not provide a definition of ‘modification’ | 2 |
| Different aftercare is required | 1 |
| **Evidence** | **42** |
| There are uncertain or changed outcomes | 28 |
| Currently being delivered within a research study | 17 |
| … within or outside of the organisation | 7 |
| … and it will be delivered/used outside of the research protocol | 13 |
| Delivery/use is prior to the commencement of a research study | 1 |
| Delivery/use has previously been halted at the trust | 1 |
| Delivery/use is to demonstrate proof of concept | 1 |
| **Economic** | **10** |
| Delivery/use has financial/resource implications | 10 |
| **External guidance** | **3** |
| Recommendations from NICE or other national/international bodies | 3 |

^1^Policies may be coded to more than one theme

NHS = National Health Service; NICE = National Institute for Health and Care Excellence; IPAC = Interventional Procedures Advisory Committee
