# Supplementary material for: Healthcare organization policy recommendations for the governance of surgical innovation: review of NHS policies
Source: Br J Surg. 2022 Jul 30;109(10):1004–12. doi: 10.1093/bjs/znac223 (PMC10364689; doi:10.1093/bjs/znac223)
Supplement: znac223_Supplementary_Data [file znac223_supplementary_data.zip › Supplementary Table 4_Final.docx]

Supplementary Table 4. Over-arching and combined over-arching themes, individual themes and sub-themes describing when new invasive procedures and devices are recommended for research ethics committee application

| **Over-arching theme**  Individual theme  Sub-theme | **Number of policies with text coded to theme, n=34^1^** |
| --- | --- |
| **External guidance**  Classified by NICE/NICE IPAC as to be delivered within research  There is no NICE or national best practice guidance | **18**  15  3 |
| **Evidence**  There is an insufficient evidence base to support delivery or use of the invasive procedure or device  … but no definition of insufficient/sufficient given  … and the required level of evidence is specified  There are uncertain or changed outcomes  … related to safety/efficacy  There is any uncertainty related to delivery/use  The invasive procedure/device is ‘original’, ‘completely new’ or ‘experimental’ (no definitions given)  Delivery/use is within a project to determine its effectiveness | **13**  6  4  2  5  3  2  4  1 |
| **Evidence *and* External guidance**  The invasive procedure/device is not established in clinical practice and delivery/use has not been notified to NICE IPAC | **2**  2 |
| **Place**  Delivery/use is for the first time anywhere | **7**  7 |
| **Place *and* Evidence**  The invasive procedure/device has not previously been performed in the organisation and is not established in clinical practice | **1**  1 |
| **Personnel**  Delivery/use requires additional training | **2**  2 |
| **Procedure**  The invasive procedure involves gene therapy | **1**  1 |

^1^Policies may be coded to more than one theme

NICE = National Institute for Health and Care Excellence; IPAC = Interventional Procedures Advisory Committee
